# Interface engineered glass fibers improve performance and durability of stabilized silty clay

**DOI:** 10.1016/j.isci.2026.116327

**Published:** 2026-06-27

**Authors:** Benhui Pang, Ran Zhang, Xin Yang, Xinyue Hou, Shanshan Tao, Zhengjun Wang

**Affiliations:** 1 School of Water Conservancy and Electric Power, Heilongjiang University, Harbin 150080, China; 2 Nanyang Yindan Irrigation District Administration, Nanyang 474150, China; 3 Heilongjiang Provincial Water Resources Research Institute, Harbin 150080, China

**Keywords:** Composite materials, Interface science, Nanomaterials, Natural material, Transportation engineering

## Abstract

Freeze-thaw cycles (FTCs) weaken stabilized subgrade soils in cold regions by promoting cracking, pore coarsening, and strength loss. In this study, glass fibers (GFs) were modified with γ-aminopropyltriethoxysilane (KH550) and epoxy resin to obtain modified glass fibers (MGFs), and nano-TiO_2_ was further anchored to obtain reinforced glass fibers (RGFs). Cement-metakaolin-stabilized silty clay (CMS) was used as the matrix to evaluate mechanical performance, permeability resistance, and freeze-thaw durability. The 28-day compressive strengths of MGF-1.25% and RGF-1.25% reached 4.25 and 4.98 MPa, increasing by 30.37% and 52.76% compared with CMS. After 20 FTCs, strength retention increased by 17.1% and 22.1%, with reduced permeability. X-ray photoelectron spectroscopy (XPS), mercury intrusion porosimetry (MIP), and scanning electron microscopy (SEM) analyses revealed stronger interfacial bonding, refined pores, and enhanced formation of hydration and polymerization products, providing a design route for durable cold-region subgrade materials in transportation infrastructure.

## Highlights

• Hierarchical interface engineering turns glass fibers into active reinforcements
• The engineered interphase enabled synergistic strengthening of silty clay
• The system shows strong potential for cold-region subgrade improvement
• XPS, MIP, XRD, FTIR, and SEM revealed coupled interface and pore evolution

## Graphical abstract

**Figure:**
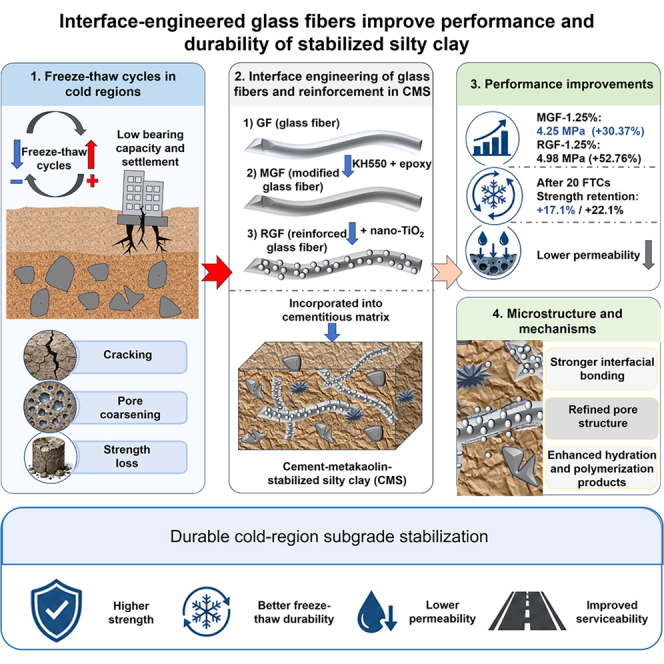

## Teaser

Composite materials; Interface science; Nanomaterials; Natural materials; Transportation engineering

## Introduction

Silty clay (SC), a widely distributed soft soil^1^ commonly found in coastal regions and alluvial plains worldwide,^2^ is frequently used as a load-bearing layer or filling material in road engineering, building foundations, and municipal infrastructure. However, it exhibits inherent shortcomings, including low natural strength and high compressibility,^3^^,^^4^ and undergoes pronounced structural degradation upon water exposure, posing substantial uncertainties and safety risks in foundation engineering.^5^ First, its loose and disordered pore structure leads to low bearing capacity and makes it highly susceptible to deformation under external loading.^6^^,^^7^ Second, its engineering behavior is strongly moisture-dependent: Water infiltration softens the soil matrix and sharply reduces strength and stiffness,^8^ whereas drying induces capillary water migration and cracking, undermining soil integrity.^9^ In addition, SC generally exhibits high permeability and water sensitivity,^10^ and under rainfall or dynamic loading, pore water pressure can accumulate rapidly, triggering slope failures, roadbed settlement, and uneven foundation deformation.^11^^,^^12^ Therefore, improving the stability and engineering performance of SC has long been a critical challenge in geotechnical and civil engineering.

**Figure 1:**
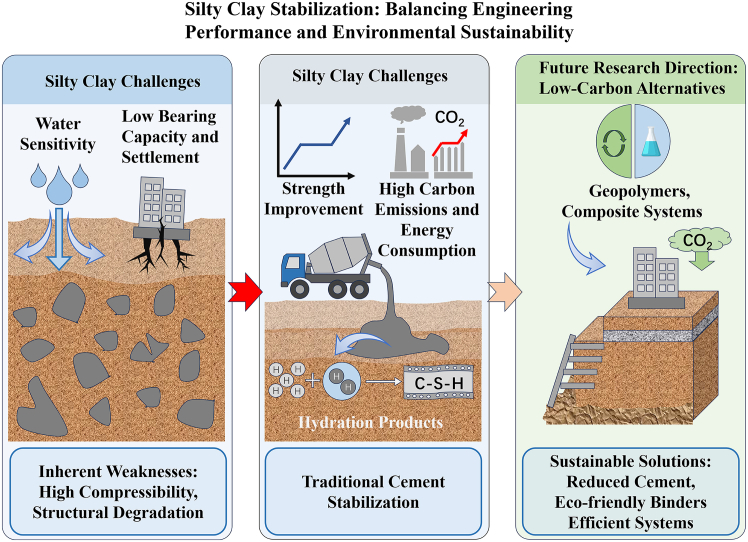
Research the background of silty clay

For soft soils, physical improvement methods such as replacement layer filling^13^ and vibratory compaction^14^ can alter soil structure, void ratio, or moisture content, thereby improving bearing capacity.^15^^,^^16^ However, these techniques offer limited effectiveness in deep soft layers and are often associated with extended construction periods. Consequently, chemical stabilization has become the predominant solution. Such methods rely on adding cementitious agents that generate new hydrates or enhance interparticle bonding to strengthen the soil matrix.^17^^,^^18^ Common chemical stabilization techniques include cement solidification, lime treatment, and geopolymer solidification. Among these, cement stabilization is the most widely adopted approach for SC due to its low cost, ease of application, and substantial strength improvement. Cement hydration generates gel-like products such as calcium silicate hydrate (C-S-H) and calcium aluminate hydrate (C-A-H),^19^ which fill pores and bind soil particles, leading to increased compressive strength, shear strength, and structural stability.^20^ Nevertheless, cement production is a major contributor to global energy consumption and carbon emissions.^21^ Recent assessments further indicate that the scale of cement-related carbon exchange is substantial. In 2023, the global annual cement CO_2_ uptake reached 0.93 Gt/yr (95% confidence interval: 0.80–1.13 Gt/yr). From 1930 to 2023, the global cumulative cement CO_2_ absorption reached 23.89 Gt (95% confidence interval: 20.47–28.74 Gt), equivalent to 52.32% of the CO_2_ process emissions from cement production during the same period.^22^ It was predicted that the annual global production of Portland cement could surpass 4.5 Gt/year by 2050 in the low-variability scenario.^23^ As carbon-neutrality goals advance, the environmental burden associated with cement-based stabilization has received growing attention. Continued reliance on high cement content inevitably intensifies emission pressures. Therefore, identifying ways to reduce cement usage, introduce low-carbon alternatives, or construct highly efficient composite stabilization systems has become a key focus in current SC improvement research. Figure 1 shows the background of engineering problems and the motivation for improvement research on SC. In this context, the partial substitution of cement by metakaolin (MK) and the achievement of favorable strength and durability within a moderate cement-content range may imply a potentially lower cement-related environmental burden, because previous studies have shown that MK can enhance durability under partial cement replacement and that minimizing binder dosage while meeting target strength generally reduces environmental impacts, although no direct CO_2_ quantification or life cycle analysis is conducted in the present study.

In recent years, geopolymers, as promising aluminosilicate gel materials with potential sustainability advantages, have attracted increasing attention in ground-improvement research.^24^^,^^25^ Geopolymers derived from alkali-activated fly ash or slag exhibit excellent mechanical properties,^26^ including high early strength and strong resistance to sulfate attack and acid corrosion.^27^^,^^28^ Their successful application in various soil types highlights their potential to overcome the limitations of traditional cementitious systems. Moreover, modern civil engineering materials are evolving beyond basic mechanical enhancement toward multifunctionality, sustainability, and intelligent performance.^29^^,^^30^ In alignment with this trend, numerous studies have incorporated fibers or nano-reinforcing phases to enhance toughness and crack resistance.^31^^,^^32^ Composite materials synergistically integrate the physical reinforcement of fibers with the chemical bonding of the matrix; the fiber bridging effect and matrix hydration jointly improve ductility and crack resistance. Glass fiber (GF), as a high-performance reinforcing material,^33^ possesses high strength, low density, and recyclability,^34^ making it advantageous for construction applications.

Zhou et al. prepared ODOPB@KH560@GF organic-modified GFs for water-based intumescent fire-resistant coatings and reported improved char-layer thermal stability and fire protection. Incorporating 5 wt % modified fibers (AL-4) reduced the peak heat release rate and total heat release by 60.5% and 58.8%, respectively.^35^ Zhang et al. demonstrated that silane-modified basalt fibers enhance geopolymer concrete (GPC) performance by improving fiber-matrix interlocking and chemical bonding, substantially increasing flexural strength.^36^ Zhang et al. further showed that graphene/graphene oxide (GO)-functionalized wood fibers reduce water absorption and porosity by 51.6% and 48.6%, while increasing compressive and flexural strengths by 13.7% and 36.3%, through improved fiber-matrix bonding and a densified interfacial transition zone (ITZ).^37^ Li et al. found that a hybrid reinforcement of 0.10% modified multi-walled carbon nanotubes (MWCNTs) and 2.00% polyvinyl alcohol (PVA) fibers significantly improved carbonation resistance and compressive strength in fly ash geopolymers.^38^ Ni et al. reported that acid-base/KH550-modified basalt fibers enhanced basalt fiber-reinforced asphalt mastic (BFRAM) performance: At 82 °C, the rutting factor increased by 2.7 times, and the glass transition temperature decreased by 4.9 °C, indicating superior high-temperature stability and low-temperature elasticity.^39^ Overall, recent studies confirm the considerable potential of fibers in cement-based materials, geopolymers, and prefabricated components. The reinforcing effect of fibers in inorganic binders is strongly governed by surface chemistry, wettability, and interfacial reactivity.^40^ However, most existing research focuses on polymer-based or geopolymer-based matrices, and comprehensive investigations into the reinforcing mechanisms and mechanical enhancement of modified fibers in soil remain limited. More specifically, most reported modification strategies still operate predominantly at a single scale. In general, silane-modified fibers mainly improve interfacial chemical affinity and bonding, whereas GO-functionalized or nanoparticle-functionalized fibers mainly enhance surface roughness, nucleation activity, or pore-filling capability.^36^^,^^37^^,^^38^^,^^39^ Although these approaches can improve fiber-matrix interaction to some extent, they usually emphasize either molecular-level coupling or nanoscale surface functionalization, rather than integrating them into a unified interfacial architecture, especially in soil reinforcement systems. As a result, improvements in interfacial chemical reactivity, stress-transfer efficiency, and interfacial stability are often not achieved simultaneously. Therefore, developing a hierarchical interfacial engineering strategy that combines chemical coupling, interfacial binding, and nano-scale structuring is essential to overcome these limitations and achieve durable interfacial performance in reinforced soils.

In this study, a hierarchical micro-nano interfacial architecture was rationally designed by integrating GFs as the reinforcing skeleton and epoxy resin as the interfacial binding phase. A KH550–epoxy layer was first constructed on the fiber surface, followed by controlled anchoring of nano-TiO_2_, forming a three-dimensional crosslinked network with increased interfacial contact sites. This engineered interface enhances chemical reactivity, nanoparticle fixation, and load transfer efficiency, thereby shifting the reinforcement mechanism from conventional physical bridging toward a synergistic chemical-physical coupling effect. Such a mechanism directly addresses long-standing challenges in stabilized soil systems, including nanoparticle agglomeration, inadequate dispersion, and weak matrix-fiber bonding. Beyond solving these system-specific problems, the proposed interfacial strategy provides a microstructural design approach that can potentially benefit a broader class of cementitious and geopolymer composites. By combining silane chemistry, hierarchical surface structuring, and nano-enabled interfacial tailoring, this work provides a pathway for controlling microstructural evolution, crack growth, and multiphase interactions in geomaterials. The ability to manipulate interfacial evolution, crack propagation, and multiphase interactions offers valuable insights for developing durable cold-region geotechnical materials and may also support future progress in multifunctional infrastructure systems, including photocatalytic geocomposites and environmental remediation matrices.

## Results and discussion

### Basic properties of silty clay

**Figure 2:**
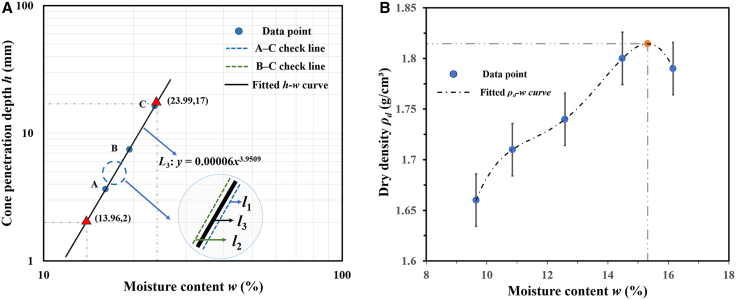
Basic index-property determination of silty clay (A) Combined liquid-plastic limit determination and fitted h-w relationship. (B) Relationship between dry density and moisture content obtained from the compaction test. Data are represented as mean ± standard deviation (SD), *n* = 3 independent measurements.

Figure 2A shows the combined liquid-plastic limit determination together with the fitted *h*-*w* relationship. The combined liquid and plastic limit determination yielded data points A (3.65, 16.1), B (7.45, 19.4), and C (16.35, 23.6). Connecting points A and C gave line *l*_1_: *y* = 0.00007*x*^3.921^, and connecting points B and C gave line *l*_2_: *y* = 0.00005*x*^4.0108^. When the cone sinking depth is 2 mm, the corresponding moisture contents for the two lines are 14.04% and 13.69%, respectively. The calculated difference between the two moisture contents is 0.35% < 2%, satisfying the acceptance criterion and indicating good consistency of the test results. The average of the two moisture contents is calculated and recorded as point D (2, 13.865). Connecting points A and D yields the *h*-*w* curve *l*_3_: *y* = 0.00006*x*^3.9509^. According to the *h*-*w* curve, when the cone penetration depth is 17 mm, the liquid limit is 23.99%; when the cone penetration depth is 2 mm, the plastic limit is 13.96%. The plasticity index, calculated using Eq. *I*_*P*_ = *w*_*L*_-*w*_*P*_, is 10.03%, classifying this SC sample as low-plasticity SC.^41^^,^^42^

The *h*-*w* curve exhibits a good power function relationship, with an exponent between approximately 3.9 and 4.0, consistent with the empirical model of moisture content controlling yield strength in fine-grained soils. This reflects the typical behavior of cohesive soils, in which increasing moisture weakens the structure and reduces yield strength. The obtained classification provides a basis for the subsequent analysis of compaction behavior and modification response.

Figure 2B shows the relationship between dry density and moisture content. The measured data exhibited a typical unimodal compaction trend. A fitted curve obtained by the least-squares method was used to estimate the optimum moisture content and the corresponding maximum dry density for subsequent specimen preparation. The fitted curve indicates that the sample reached its maximum dry density at a moisture content of 15.3%, with a corresponding maximum dry density of 1.81 g/cm^3 43^. In the low moisture content stage, soil particles are difficult to lubricate, and the compaction work mainly overcomes inter-particle friction, resulting in a rapid increase in dry density with increasing moisture content. Near the optimum moisture content, moisture provides a lubricating effect and partially fills the pores, causing the particles to gradually become more densely packed. At higher moisture contents, free water increases, making it difficult for pore water to be squeezed out. Instead, it occupies effective compaction space, leading to a decrease in dry density.

This parameter provides a useful basis for determining the optimum moisture content control window and predicting the compaction performance of fill.

### Optimization of reinforced glass fibers

**Figure 3:**
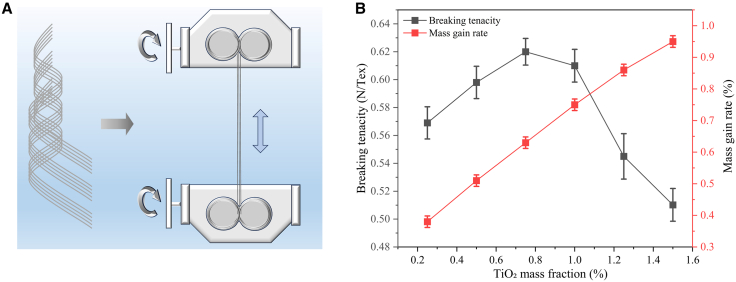
Nano-TiO_2_ dosage controls the tensile performance and surface deposition behavior of reinforced glass fibers (A) Schematic diagram of the fiber tensile test. (B) Variation of breaking tenacity and mass gain rate of RGF with nano-TiO_2_ mass fraction. Data are represented as mean ± SD, *n* = 10 fibers.

Figure 3A shows a schematic diagram of the tensile test for the breaking tenacity of GF, and Figure 3B shows the curves of breaking tenacity and mass gain rate of GF. As shown in the figure, the breaking strength of reinforced glass fiber (RGF) exhibits a typical first-increasing-then-decreasing trend with increasing nano-TiO_2_ content. This mechanical behavior reflects the competition between effective surface functionalization and particle agglomeration, which jointly govern the interfacial stress transfer efficiency.

When the nano-TiO_2_ content increases from 0 to 0.75 wt %, the breaking strength of RGF increases continuously, reaching the maximum value of 0.62 N/Tex at 0.75 wt % nano-TiO_2_. At this point, the TiO_2_ nanoparticles are uniformly anchored to the GF surface in two ways. First, the surface hydroxyl groups of the nano-TiO_2_ underwent condensation reactions with the ring-opened hydroxyl groups of the GF or epoxy resin, forming Ti–O–Si and Ti–O–C covalent bonds. Through this bridging effect, the nano-TiO_2_ and RGF are chemically linked, which strengthens interfacial bonding and improves the mechanical integrity of the modified fiber. Second, the three-dimensional network formed after the epoxy resin cures physically encapsulated the nano-TiO_2_, resulting in a moderate increase in surface roughness, effective contact area, and surface energy. This enhances the frictional resistance between the fiber surface and the tensile clamping system, improving shear transfer during the tensile process. The micro-anchoring effect caused by well-dispersed nanoparticles also promotes uniform stress distribution along the fiber axis, significantly reducing stress concentration and delaying micro-crack propagation, thereby enhancing fiber integrity under tensile loading.

However, when the nano-TiO_2_ content exceeds 0.75 wt %, the breaking strength begins to decline. This reduction is attributed to the onset of nanoparticle agglomeration, which creates local defects or weak points on the fiber surface. Agglomerated nano-TiO_2_ clusters act as mechanical discontinuities, deteriorating the interfacial compatibility between the GF and the testing apparatus and inducing stress localization during tensile deformation. In addition, excessive nano-TiO_2_ weakens the effective bonding due to poor wettability and insufficient surface activation, which leads to slippage and early debonding under tensile force, eventually causing premature fiber fracture.^43^^,^^44^

The mass gain curve shows that the mass gain rate increases monotonically with increasing nano-TiO_2_ content. However, this trend does not correspond to a simultaneous strengthening of mechanical performance. This suggests that beyond the optimal content, the additional TiO_2_ contributes primarily to non-functional deposition or surface accumulation, rather than forming effective interfacial adhesion sites.

The combination of the breaking strength curve and the mass gain curve clearly reveals a nonlinear structure-property relationship, highlighting that the optimal combination between effective surface modification and structural integrity is achieved at 0.75 wt % nano-TiO_2_. Therefore, RGF prepared with 0.75 wt % nano-TiO_2_ was selected as the representative reinforced-fiber formulation for subsequent soil reinforcement experiments. It should be noted, however, that the present study does not directly verify whether 0.75 wt % is also the globally optimal nano-TiO_2_ dosage in the soil composite system.

### Optimization of cement-metakaolin-stabilized silty clay (CMS)

A two-way analysis of variance (ANOVA) was conducted to evaluate the main effects of cement content (A) and MK content (B), as well as their interaction (A×B), on the shear strength of the composite soil. As summarized in Table 1, both factors exhibit highly significant influences, with *p* values well below 0.001 for the main effects and below 0.01 for the interaction term. These results indicate that shear strength is controlled by the combined effects of cement and MK rather than by either factor alone.

**Table 1:** Two-way analysis of variance table

Variance	df	SS	MS	F	*p* value
A	2	1.713	0.8566	19.1	<0.001
B	2	0.783	0.3916	8.69	<0.001
A×B	4	0.943	0.2357	5.23	<0.01
Error	18	0.811	0.0451	–	–
Total	26	4.250	–	–	–

Two-way analysis of variance results for the effects of cement and metakaolin on shear strength. A indicates cement content; B indicates metakaolin content; A × B indicates the interaction between cement and metakaolin. df, degrees of freedom; SS, sum of squares; MS, mean square. *p* values indicate the statistical significance of each factor and its interaction.

The sum of squares attributed to cement content, SS(A) = 1.713, accounts for 40.3% of the total variance, highlighting cement as the dominant contributor to strength development. This is consistent with the dominant role of cement hydration products in forming the early load-bearing skeleton. The interaction term exhibits a substantial contribution (SS(A×B) = 0.943), indicating that the strengthening contribution of MK is critically dependent on the availability of Ca(OH)_2_ released during cement hydration. This result reflects the pozzolanic nature of MK, whose strengthening effect depends on sufficient Ca(OH)_2_ for the formation of secondary C-S-H.

The F-statistics for cement and MK are 19.1 and 8.69 times the unit random error, respectively, strong effect sizes. The interaction term also yields a statistically significant F-value (*p* < 0.01), confirming a non-additive and synergistic influence of the two materials on shear behavior.^45^ These findings collectively indicate that optimizing the composite soil requires a coordinated, rather than an independent, adjustment of cement and MK contents.

To rationalize the selection of a practically applicable mix ratio, three quantitative functions were constructed: performance function *Z*_*strength*_ = *f*(*C*,*MK*), cost function *Z*_*cost*_ = 0.38*C* + 2.7 *MK*, and cost-effectiveness index function $Z_{ratio}=\frac{Z_{strength}}{Z_{cost}}$ serving as an engineering decision metric that quantifies structural benefit per unit expenditure.

**Figure 4:**
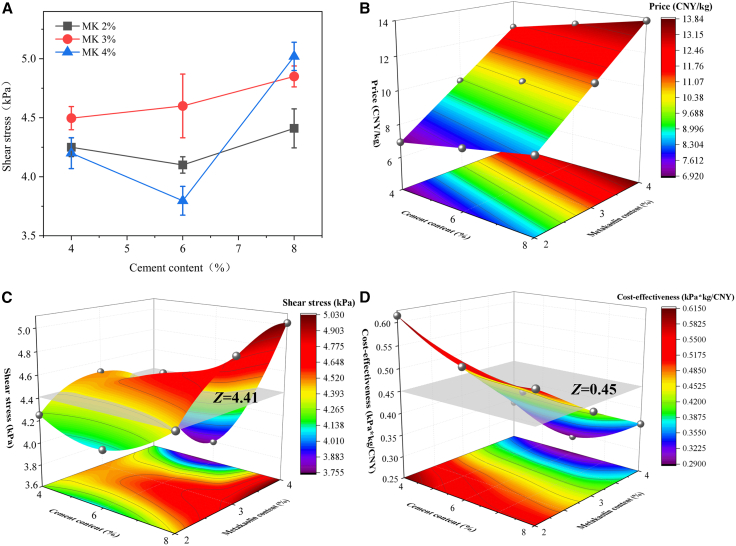
Cement-metakaolin proportioning balances mechanical performance, material cost, and cost-effectiveness in CMS optimization (A) Cement-metakaolin interaction plot. (B) Cost surface. (C) Performance surface. (D) Cost-performance ratio surface. Data are represented as mean ± SD, *n* = 3 independent specimens.

Figure 4A shows the interaction effect between cement (C) and MK. The pronounced change in response trends across different MK levels indicates a clear and significant interaction. The simple-effect analysis reveals that the contribution of MK is strongly dependent on the baseline cement content. At lower cement dosages, MK exhibits a peak-strength behavior, with the mix proportion CMS-6-3 yielding the most favorable enhancement. However, as cement content increases, the beneficial effect of MK becomes progressively more pronounced, showing a monotonic strengthening trend with increasing MK dosage. Overall, the combined analysis suggests that the mixture containing 8% cement and 4% MK offers the most effective reinforcement among the tested levels.

Figure 4B shows the cost surface plots for various cement-MK blending ratios. The surface shows a generally monotonic increase in cost with increasing contents of both constituents; however, the noticeably sharper curvature along the MK axis indicates its substantially higher marginal cost. This result highlights the need to optimize the MK dosage to balance performance and cost. More importantly, by quantitatively revealing the nonlinear cost sensitivity of the two binders, this analysis provides a practical decision-making basis for mix-design optimization in resource-constrained cold-region projects, enabling engineers to achieve targeted mechanical and durability improvements without incurring unnecessary material expenses.

Figure 4C shows the performance surface plots for different cement-MK proportions. The average technical performance was 4.41 kPa, which was adopted as the threshold to identify mix proportions capable of delivering medium-to-high mechanical improvement. As shown in Figure 4C, the combinations CMS-4-3, CMS-6-3, CMS-8-2, CMS-8-3, and CMS-8-4 all satisfied the required performance level. Among them, CMS-8-3 (4.85 kPa) and CMS-8-4 (5.02 kPa) exhibited particularly notable enhancements. These pronounced gains can be attributed to the intensified formation of secondary C-S-H gels generated through the pozzolanic reaction between MK and Ca(OH)_2_, which strengthens interparticle bonding and enhances the structural skeleton. The performance surface therefore provides a quantitative basis for identifying efficient binder combinations.

Figure 4D shows the cost-effectiveness curve. The average cost-effectiveness for different proportions of cement and MK was 0.45 kPa/yuan. An economic threshold of 0.45 kPa/yuan was adopted to ensure economic feasibility. As shown in Figure 4D, the four combinations CMS-4-2, CMS-4-3, CMS-6-2, and CMS-8-2 met the performance requirements. With increasing cement and MK content, the cost-effectiveness gradually decreased. When the MK content remained constant, the impact of increasing cement content on cost-effectiveness decreased; when the cement content remained constant, the impact of MK content on cost-effectiveness remained essentially unchanged. This is because MK, as a pozzolanic material, lacks inherent cementitious properties and requires a pozzolanic reaction with Ca(OH)_2_, a byproduct of cement hydration, to generate calcium silicate hydration products with strength.^46^^,^^47^ With a fixed MK content, higher cement content results in more Ca(OH)_2_ produced during cement hydration. Initially, the cost-effectiveness increases significantly with increasing cement content. However, as cement content increases to a certain level, excess Ca(OH)_2_ lacks sufficient MK to react and exists only in a weak crystalline form, contributing little to strength. At this point, the synergistic strengthening effect of MK decreases, strength increase slows, and thus the decrease in cost-effectiveness slows. Cement hydration is difficult to complete within seven days. When cement content remains constant, the pozzolanic reaction is difficult to effectively improve, the cost-effectiveness gradually decreases with increasing MK content. Overall, the cost-effectiveness analysis clarifies the diminishing returns associated with excessive binder additions and highlights the importance of balancing cement hydration and pozzolanic activity to achieve economically optimized mix designs.

Looking at the performance surface and cost-effectiveness curves, the compliance areas of the two standards show significant overlap in the mix proportions: 4% cement, 3% MK, and 8% cement, 2% MK. Meanwhile, when cement is at 8% and MK at 3%, the cost-effectiveness is very close to the economic threshold, but the performance is 10% higher than the technical threshold of 4.4 kPa, providing a more favorable balance between mechanical robustness and cost efficiency. After comprehensive consideration, 8% cement and 3% MK were selected as the most suitable binder combination for subsequent testing. It should be noted that this selection was based on early-age shear performance together with cost-effectiveness, and therefore served as a practical screening step rather than a full multi-objective optimization of long-term behavior; the selected binder system was subsequently validated by 28-day compressive strength, permeability, and freeze-thaw resistance tests. Subsequent compaction testing confirmed an optimum moisture content of 18.1%, further validating the engineering applicability of the optimized cement-MK mix.

### Mechanical performance of reinforced stabilized silty clay

**Figure 5:**
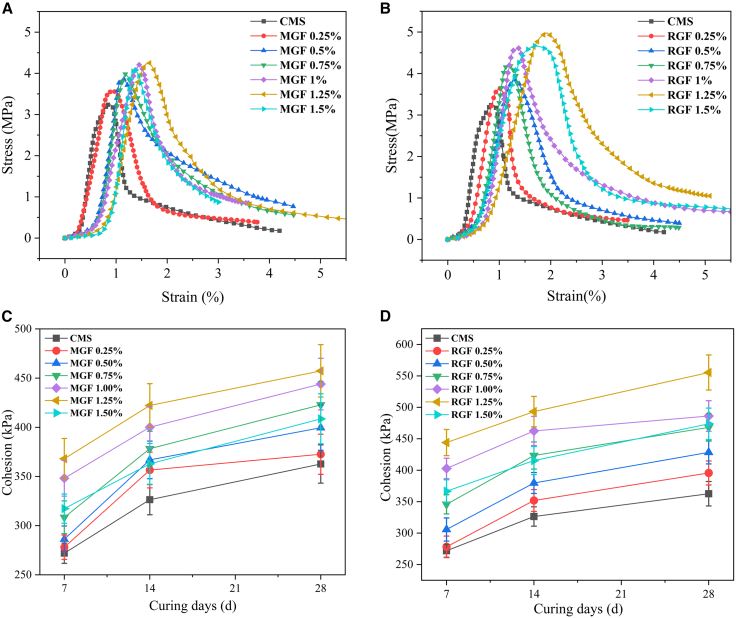
Mechanical properties of CMS, MGF-modified, and RGF-modified specimens (A) Stress-strain curves of CMS and MGF-modified specimens at 28 days. (B) Stress-strain curves of CMS and RGF-modified specimens at 28 days. (C) Cohesion of CMS and MGF-modified specimens at different curing ages. (D) Cohesion of CMS and RGF-modified specimens at different curing ages. Error bars in (C) and (D) represent mean ± SD, *n* = 3 independent specimens.

Figures 5A and 5B show the 28-day compressive strength curves of the tested soil samples. The compressive strength of the CMS specimen reached 3.26 MPa, far exceeding that of the untreated SC (0.21 MPa). This significant improvement stems from the formation of a continuous cement skeleton, in which primary hydration products—C-S-H gel and crystalline Ca(OH)_2_—bridge and fix loose soil particles, thereby enhancing the soil’s bearing capacity and stiffness.^48^ In the mixed cement-MK system, active amorphous SiO_2_ and Al_2_O_3_ undergo a more vigorous pozzolanic reaction with Ca(OH)_2_, generating secondary C-S-H and C-A-H gels.^49^^,^^50^ These secondary gels refine the pore network, enhance interparticle contact, and increase the volume fraction of the cohesive phase, thereby significantly improving mechanical strength.

The 28-day peak compressive strengths of soil samples with MGF contents of 0.25%, 0.50%, 0.75%, 1.00%, 1.25%, and 1.50% were 3.32 MPa, 3.86 MPa, 3.98 MPa, 4.20 MPa, 4.25 MPa, and 4.10 MPa, respectively. The 28-day peak compressive strengths of soil samples with RGF contents of 0.25%, 0.50%, 0.75%, 1.00%, 1.25%, and 1.50% were 3.47 MPa, 4.00 MPa, 4.42 MPa, 4.61 MPa, 4.98 MPa, and 4.67 MPa, respectively. The peak unconfined compressive strength (UCS) values of MGF-1.25% and RGF-1.25% were 4.25 ± 0.20 MPa and 4.98 ± 0.29 MPa, respectively (*n* = 3), representing increases of 30.37% and 52.76% compared to CMS. As the GF content increases, the compressive strength of the soil exhibits a trend of first increasing and then decreasing; the higher the soil strength, the higher its peak strain.^51^ Compared with previously reported fiber-reinforced cementitious and geopolymer systems, the compressive-strength enhancement observed in the present study appears to be more pronounced. For example, in GF-reinforced concrete, fiber incorporation alone increased the 28-day compressive strength by only about 10%,^52^ whereas the improvement in freeze-thaw durability was mainly associated with pozzolan-induced matrix densification.^53^ Likewise, red mud-slag-based fiber-reinforced geopolymers showed an initial increase followed by a decrease in strength with increasing fiber content, indicating that excessive fiber incorporation may introduce defects and compromise mechanical performance. Although the matrix systems are not identical, these findings suggest that conventional fiber reinforcement is governed primarily by crack bridging and matrix densification. By contrast, the present system benefits from an engineered interphase that more effectively couples chemical bonding, mechanical interlocking, and pore refinement.

It is also noteworthy that comparable strength levels reported in previous stabilized-soil studies often required higher cement contents. For example, a modified rammed-earth system reached 4.47 MPa at 15% cement, and a tropical expansive clay reached 4.547 MPa at 11% cement.^54^ Although the material systems are not identical, these comparisons suggest that the present cement-MK system achieves favorable mechanical performance with a lower dependence on cement input. Since cement production is a major source of carbon emissions in binder-based soil stabilization, the favorable strength achieved here with a lower cement dosage may also imply a potentially lower cement-related carbon burden at the mix-design level, although no direct CO_2_ quantification or life cycle analysis was conducted in the present study.

At the macroscopic scale, the dispersed fibers form a quasi-isotropic reinforcing network that restrains the initiation and opening of microcracks. Under compressive loading, the fibers provide a “bridging–pull-out–slip” energy dissipation mechanism, effectively delaying catastrophic fracture and increasing peak strain capacity. The optimized fiber content ensures uniform stress transfer paths and minimizes localized discontinuities that would otherwise act as preferential crack propagation channels.

At the microscopic and interfacial scales, after surface modification, the GFs and hydrolyzed KH550 are firmly linked by Si-*O*-Si covalent bonds, thus anchoring the organic ends to the inorganic fiber surface. The amino groups at the organic ends undergo ring-opening reactions with the epoxy resin, forming strong chemical bonds. The hydroxyl groups formed after epoxy ring opening can condense with active hydroxyl groups on the inorganic fiber surface. This reaction forms stable C–O–Si or C–O–Al bonds and chemically bridges the fiber, epoxy phase, and matrix. Building upon this foundation, the nano-TiO_2_ added to RGF can fill micropores, reducing porosity. Furthermore, the hydroxyl groups on its surface promote accelerated nucleation, enhancing early compressive strength.^55^

For RGF, the nano-TiO_2_ coating introduces an additional hierarchical strengthening mechanism. The nanoscale particles effectively fill microvoids on both the fiber surface and the surrounding matrix, contributing to pore refinement and reduced local porosity. The abundant surface hydroxyl groups on nano-TiO_2_ act as heterogeneous nucleation sites for early-age C-S-H formation, accelerating hydration kinetics and producing a denser, more cohesive ITZ. Moreover, the nano-TiO_2_ nanoparticles increase surface roughness and frictional resistance, thereby enhancing mechanical interlocking and promoting a more stable fiber-pull-out behavior.

The combined effect of chemical coupling, mechanical interlocking, and nanoparticle-induced densification results in the formation of a three-dimensional cross-linked microstructure. This hybrid interphase not only increases frictional resistance and surface adhesion but also facilitates more efficient stress transfer between the matrix and the reinforcing phase. Consequently, at the optimal fiber dosage, a pronounced synergistic strengthening effect is achieved, reflected in the significant gains in compressive strength and ductility of the reinforced soil.

The internal friction angle (φ) of both the MGF-1.25% (35.81°) and RGF-1.25% (38.14°) samples increased compared with the CMS sample (31.78°). This is attributed to the addition of GFs, which enhances the chemical reaction and mechanical interlocking of C-S-H and C-A-H gels, thereby increasing the friction angle of the matrix. This observation aligns with contact mechanics theory, whereby an increase in effective roughness density improves shear resistance at the interface. Moreover, the RGF sample exhibited a higher φ value than the MGF sample. This can be attributed to nano-TiO_2_ on the fiber surface, which fills interfacial micropores, increases the effective contact area, accelerates early C-S-H precipitation,^56^^,^^57^ and promotes the formation of a rigid fiber-matrix ITZ.^58^^,^^59^ Together, these effects enhance the frictional component of shear resistance.

Figures 5C and 5D show the cohesion variation curves, revealing the different shear strengthening mechanisms induced by fiber modification. The data indicate that the cohesion of the CMS, MGF, and RGF samples all increased with prolonged curing time, reflecting the gradual formation of cementitious products and the compaction of the soil skeleton. For the MGF and RGF samples, cohesion initially increased, then decreased as fiber content increased. After 28 days of curing, the cohesion of the MGF-1.25% and RGF-1.25% samples reached 457.35 kPa and 555.45 kPa, respectively. Compared with that of CMS (362.67 kPa), their shear strength was significantly improved. Previous studies on fiber-reinforced soils subjected to freeze-thaw cycles (FTCs) indicate that strength degradation is governed by the progressive weakening of the soil matrix, the fibers, and, most critically, the fiber-soil interface. Freeze-thaw cycling disrupts the surrounding soil structure, reduces effective contact, and weakens both interfacial adhesion and frictional resistance.^60^ In basalt fiber-reinforced cemented silty sand, the strengthening effect is mainly attributed to the combined action of cementation and fiber bridging. By contrast, the present system improves both the frictional and cohesive components of shear strength through hierarchical interfacial engineering. The KH550-epoxy-TiO_2_ interphase enhances interfacial adhesion, stabilizes stress transfer, and mitigates freeze-thaw-induced interfacial deterioration, which is consistent with the higher φ and c values observed for the RGF specimens.

This enhancement can be attributed to the following interrelated mechanisms. First, the three-dimensional cross-linked network is formed through Si-*O*-Si covalent bonds that anchor the silane coupling agent KH550 to the fiber substrate; Epoxy-amine cross-linked networks that establish durable organic-inorganic hybrid interfaces; and C-*O*-Si and C-*O*-Al bridges that chemically link fibers with surrounding hydration products. These reactions collectively yield a three-dimensional cross-linked interphase, which improves adhesive strength and facilitates continuous stress transfer across potential failure planes. Second, during shear deformation, fibers contribute to energy dissipation through mechanisms such as fiber pull-out, rupture, debonding, and interfacial sliding.^61^^,^^62^ These processes delay the initiation and propagation of macroscopic cracks. The thicker and stiffer ITZ in TiO_2_-coated RGF further enhances mechanical interlocking, resulting in greater cohesion improvement. Third, nano-TiO_2_ refines the microstructure by filling nano-to micro-scale pores, promoting C-S-H nucleation, and increasing the density of the ITZ. These effects collectively improve the continuity and integrity of the cementitious matrix, thereby elevating the cohesive component of shear strength.

### Durability test results

#### Permeability behavior

**Figure 6:**
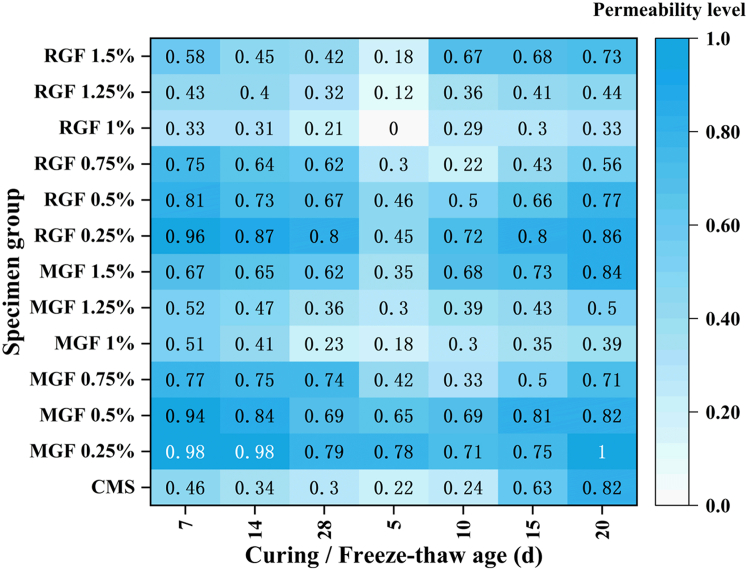
Heatmap of linearly normalized permeability levels of different specimen groups at different curing/freeze-thaw ages

Durability tests included variable head permeability tests and FTC tests. Figure 6 is a heatmap of the permeability coefficient after linear normalization. The data in the figures show that the impermeability of each group initially increases and then decreases. During curing, the gel formed by cement hydration and the pozzolanic reaction of MK continuously refines the pores, making the structure denser. In the early stages of FTCs, the impermeability slowly increases because the strengthening effect from continuous hydration outweighs the negative impact of FTCs. However, with the increase in the number of FTCs, the performance significantly decreases, and the freeze-thaw damage effect begins to dominate and accumulate. In each FTC, external liquid water seeps into the material’s internal pores, and its freezing generates enormous expansion pressure, repeatedly acting on the pore walls. These micro-damages continuously initiate, expand, and connect, eventually forming a network of interconnected macroscopic cracks, providing convenient channels for water penetration, thus continuously reducing the impermeability.^63^^,^^64^ Overall, the absolute permeability coefficient follows the order RGF < MGF < CMS < SC.

At low fiber content, it is difficult to form an effective three-dimensional network, and its crack-resistant effect is very limited, becoming a defect or stress concentration point in the material, thus inducing microcracks. As the fiber content increases, approaching the critical volumetric content, the three-dimensional fiber network gradually forms. The bridging and crack-resistant effects of the fibers are fully utilized, effectively suppressing plastic shrinkage and drying shrinkage cracks, resulting in a continuous improvement in impermeability. Beyond the optimal content, fiber agglomeration and increased porosity introduce permeation channels, thus causing a decline in impermeability.

Experimental data show that the permeability coefficient of SC is 1.68 × 10^−7^ cm/s, while that of CMS is 4.89 × 10^−8^ cm/s, representing a 71.25% reduction in permeability coefficient compared to SC. The optimal dosage of modified glass fiber (MGF) is 1%. The permeability coefficients of MGF and RGF samples are 3.35 × 10^−8^ cm/s and 3.02 × 10^−8^ cm/s, respectively, representing reductions of 31.5% and 38.2% in permeability coefficient compared to CMS. Relative to SC, the total reductions in permeability coefficient for MGF and RGF are approximately 80.1% and 82.0%, respectively. Compared with MGF, RGF exhibits a further 9.9% reduction in permeability coefficient. MGF is randomly distributed within the soil, forming a three-dimensional network structure that effectively prevents the generation and development of seepage cracks, thus exhibiting superior impermeability compared to CMS. The extremely small nano-TiO_2_ particles in RGF can fill the nanoscale pores in the cement hydration products and fiber-matrix interface region, making the material’s microstructure more compact, fundamentally reducing porosity, and increasing the tortuosity of the seepage path. This synergistic effect of fiber crack prevention and nano-filling results in optimal impermeability performance.

#### Freeze-thaw resistance

**Figure 7:**
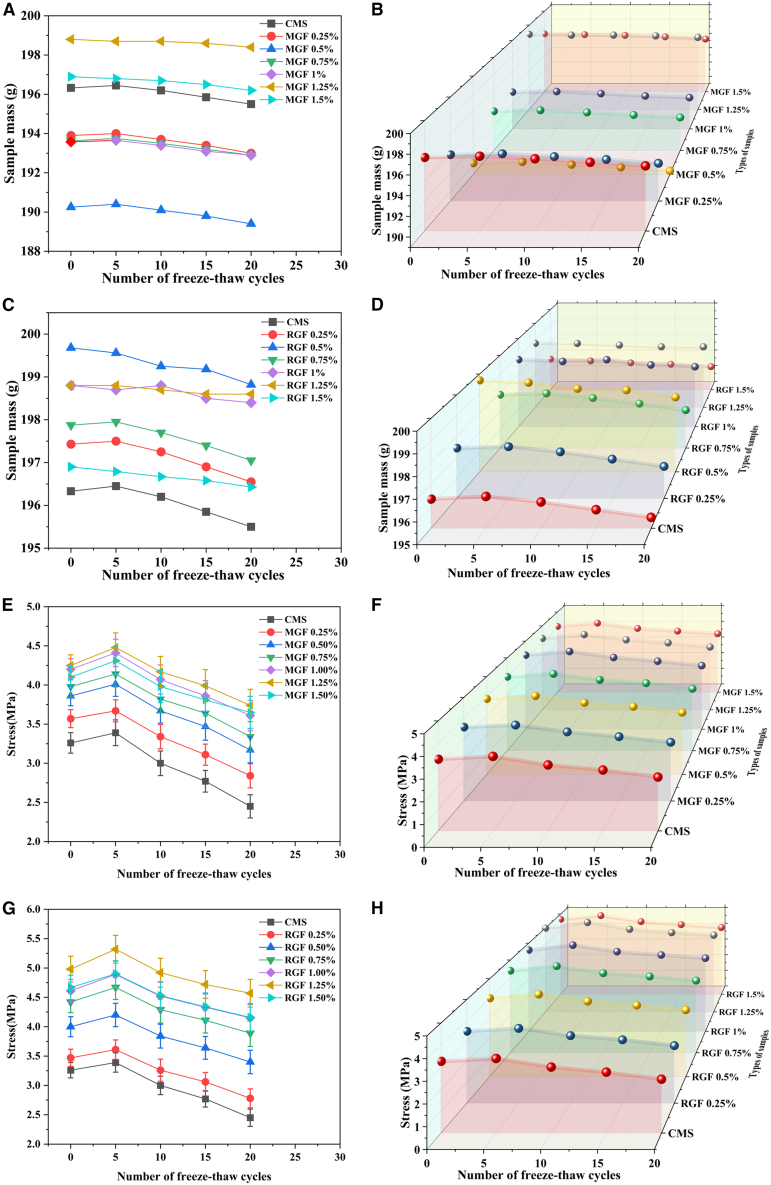
Freeze-thaw durability of CMS, MGF-modified, and RGF-modified specimens (A–D) Variation in sample mass during freeze-thaw cycles. (E–H) Evolution of residual compressive strength under freeze-thaw cycles. Error bars in (E) and (G) represent mean ± SD, *n* = 3 independent specimens.

Figures 7A–7D illustrate the variation in sample mass during freeze-thaw cycling. As the number of cycles increased, all samples exhibited varying degrees of mass loss and strength degradation. The MGF and RGF specimens showed significantly better performance than the CMS group in both mass retention and residual strength. This improvement is mainly attributed to the crack-bridging and stress-dispersing effects of the fibers. Under freeze-thaw action, the fibers restrain crack initiation and propagation, thereby reducing peeling, surface spalling, and associated mass loss. In addition, the epoxy resin coating improves interfacial stability during freeze-thaw cycling, suppresses interfacial debonding, and helps maintain a higher residual strength than CMS.

Figures 7E–7H show the evolution of residual compressive strength under FTCs. The results indicate that both MGF and RGF specimens with a fiber mass fraction of 1.25% exhibited the lowest mass loss and the smallest reduction in mechanical properties. After 20 FTCs, the absolute residual compressive strength of the CMS sample was 2.45 MPa. In comparison, the residual strengths of MGF-1.25% and RGF-1.25% reached 3.74 MPa and 4.57 MPa, respectively, corresponding to strength retention rates that were 17.1% and 22.1% higher than those of CMS. Compared with previous studies on freeze-thaw-affected fiber-reinforced geomaterials, the present system demonstrates better retention of residual strength. In basalt fiber-reinforced cemented silty sand, repeated freeze-thaw cycling still led to a stabilized strength-loss level of approximately 30%, although the combined use of cement and fibers outperformed either treatment alone.^65^ In red mud-slag-based fiber-reinforced geopolymers, freeze-thaw resistance was highly sensitive to fiber type, length, and dosage, and progressive crack development and mass loss were still observed during cyclic exposure.^66^ In contrast, the present RGF system more effectively preserves interfacial integrity through chemical coupling, epoxy-mediated bonding, and nano-TiO_2_-induced densification, thereby resulting in lower mass loss and higher residual strength.

The data indicate that the RGF samples exhibited the lowest mass loss and superior strength retention. Although only one nano-TiO_2_ dosage (0.75 wt %) was adopted in the soil composite tests, the consistently better performance of RGF than MGF in strength, impermeability, and freeze-thaw resistance provides indirect evidence that this dosage is effective for interfacial enhancement. Nevertheless, the present results should be regarded as a validation of the interface-enhancement effect rather than a definitive optimization of nano-TiO_2_ dosage in the soil composite system. This durability improvement is mainly attributed to better crack restraint, lower capillary connectivity, and improved interfacial stability during freeze-thaw cycling. Both MGF and RGF specimens showed a trend of performance improvement followed by deterioration as fiber content increased. This is because excessive fiber dosage hinders uniform dispersion within the matrix, leading to fiber agglomeration and increased porosity. The resulting defects weaken the fiber-matrix bonding and reduce the overall reinforcement efficiency.

#### Mercury intrusion porosimetry (MIP) results

**Figure 8:**
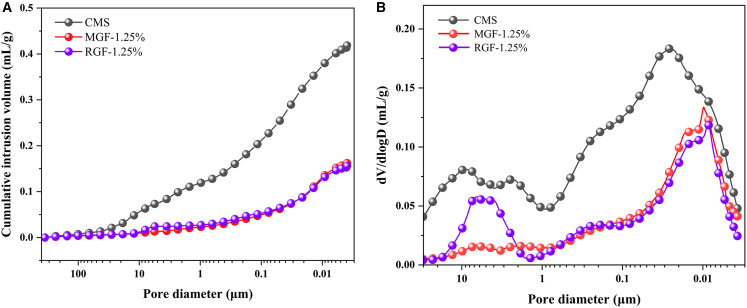
Mercury intrusion porosimetry results of CMS, MGF-1.25%, and RGF-1.25% after 20 freeze-thaw cycles: (A) cumulative intrusion volume versus pore diameter; (B) pore size distribution expressed as dV/dlogD versus pore diameter

To further clarify the durability difference among CMS, MGF-1.25%, and RGF-1.25% after freeze-thaw attack, mercury intrusion porosimetry (MIP) was used to quantify the pore-structure evolution of the specimens after 20 FTCs. The corresponding cumulative intrusion curves and pore-size distribution curves are shown in Figures 8A and 8B, and the characteristic parameters are summarized in Table 2.

**Table 2:** MIP parameters of CMS, MGF-1.25%, and RGF-1.25% after 20 freeze-thaw cycles

Sample	Total intrusion volume(mL/g)	Porosity (%)	Median pore diameter (volume) (μm)	Average pore diameter, 4 V/A(μm)
CMS	0.4154	33.32	0.10702	0.03163
MGF-1.25%	0.1615	30.81	0.03017	0.01958
RGF-1.25%	0.1538	27.55	0.02661	0.01815

MIP parameters of CMS, MGF-1.25%, and RGF-1.25% after 20 freeze-thaw cycles. MIP, mercury intrusion porosimetry; CMS, cement-metakaolin-stabilized silty clay; MGF, modified glass fiber; RGF, reinforced glass fiber. Total intrusion volume, porosity, median pore diameter, and average pore diameter were used to describe the accessible pore structure after freeze-thaw cycling. Average pore diameter is expressed as 4 V/A.

As shown in Table 2 and Figure 8A, CMS exhibits the highest total intrusion volume (0.4154 mL/g), porosity (33.32%), median pore diameter (volume) (0.10702 μm), and average pore diameter (4 V/A) (0.03163 μm), indicating that repeated FTCs generated a relatively open and coarse pore network in the cement-MK-stabilized matrix. In contrast, both MGF-1.25% and RGF-1.25% show markedly lower total intrusion volume and porosity than CMS. The total intrusion volumes for MGF-1.25% and RGF-1.25% decrease to 0.1615 and 0.1538 mL/g, respectively, while the corresponding porosities decrease to 30.81% and 27.55%. Moreover, the median pore diameter and average pore diameter further decrease from 0.03017 to 0.01958 μm in MGF-1.25% to 0.02661 and 0.01815 μm in RGF-1.25%. These results indicate that fiber incorporation effectively restrained freeze-thaw-induced pore development, with RGF showing the strongest inhibition effect.

Figure 8B further shows that CMS presents a broader coarse-pore feature in the larger-pore range together with a higher intrusion intensity over the tested pore-size range, reflecting the coexistence of relatively large freeze-thaw-induced pores/cracks and connected pore throats. By contrast, the pore-size distribution curves of MGF-1.25% and RGF-1.25% show lower intrusion intensity and a weaker coarse-pore feature, suggesting that the modified fibers suppressed pore coarsening during freeze-thaw cycling and reduced the accessibility of the pore network. Among the modified specimens, RGF-1.25% exhibits the lowest cumulative intrusion volume, porosity, median pore diameter, and average pore diameter, indicating the strongest pore-structure refinement after 20 FTCs.

As shown in Figure 6, the permeability coefficients of MGF and RGF are lower than that of CMS, while Figure 7 shows that MGF-1.25% and RGF-1.25% retain higher residual strength after 20 FTCs. The MIP results indicate that these macroscopic improvements are closely related to restrained freeze-thaw-induced pore coarsening, reduced capillary continuity, and a denser accessible pore system. In particular, the lower accessible pore volume, porosity, and characteristic pore diameters of RGF are consistent with its lower permeability and higher residual strength after FTCs. This interpretation is also consistent with the interfacial stabilization discussed in Section 2.4 and with the X-ray photoelectron spectroscopy (XPS) evidence presented in Section 2.6.3.

### Microstructural and interfacial characterization

#### X-ray diffraction (XRD) analysis

**Figure 9:**
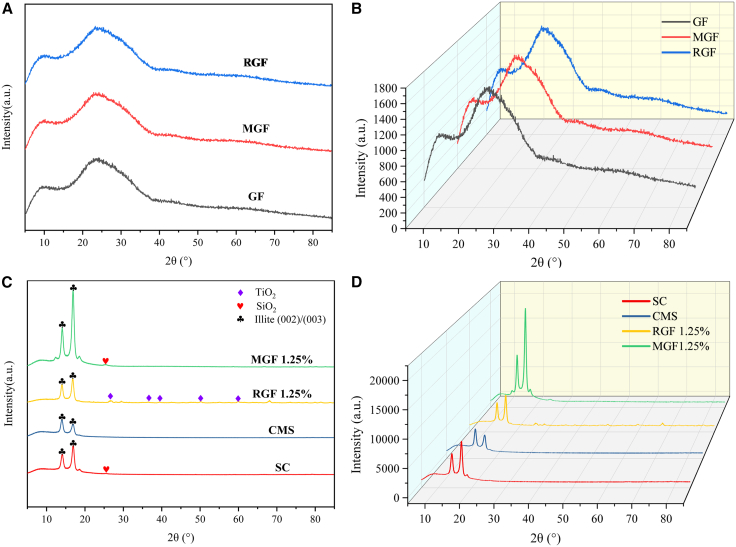
XRD patterns reveal the mineralogical evolution of glass fibers and stabilized silty clay systems after fiber modification and matrix stabilization (A and B) XRD patterns of different glass fibers. (C and D) XRD patterns of different samples.

Figures 9A and 9B show the X-ray diffraction (XRD) characteristic spectra of GFs. All samples display broad diffuse scattering humps within 2θ = 20°–30°, characteristic of amorphous aluminosilicate networks and corresponding to the first sharp diffraction peak (FSDP) commonly observed in disordered silicate systems.^67^ Compared with pristine GF, the MGF spectrum exhibits fewer micro-fluctuations and a smoother baseline, indicating reduced network heterogeneity and suppressed diffraction noise. This can be attributed to the silanization reaction between KH550 and surface -Si-OH groups, which forms a covalently anchored Si-*O*-Si polysiloxane network. The surface condensation polymerization introduces a more uniform arrangement of Si-*O*-Si linkages, enhancing the short-range order. Additionally, the KH550-derived interfacial coating forms a temporary physical cross-linked network, involving hydrogen bonding, van der Waals interactions, and partially condensed siloxane clusters. These microdomains reduce local structural fluctuations, giving rise to the smoother MGF background and indicating a shift toward higher network packing density. The RGF spectra resemble those of MGF. Although the nano-TiO_2_ content is low, preventing distinct rutile or anatase reflections at this stage, the TiO_2_ nanoparticles enhance surface energy and promote local structural rearrangement, resulting in strengthened local order without forming long-range crystalline domains.

Figures 9C and 9D show the XRD characteristic spectra of different samples. The XRD pattern of SC shows a weak, diffuse peak at 2θ = 8.766°, with a broad, low, and asymmetrical peak shape, indicating a low interlayer sequence.^68^^,^^69^ This suggests the presence of illite. The peak shift and relatively large height and width suggest the possible presence of a small amount of illite-saturated mixed-layer minerals. Two strong diffraction peaks are present simultaneously at 2θ = 14.005° and 2θ = 16.853°.^70^ This shows a clear overlap with illite, possibly indicating a kaolinite superposition zone. A weak, diffuse peak near 25.584° suggests the possible presence of free quartz (Qtz).^71^^,^^72^ Diffraction peaks at 18.576° and high-angle weak peaks at 48.856°, 57.738°, 66.618°, and 72.238° confirm the presence of Qtz. Overall, SC is a layered silicate dominated by illite, with Qtz as a secondary mineral phase and possible minor amounts of kaolinite and illite-saturated mixed-layer minerals.

The XRD pattern of CMS shows a diffuse peak at 8.763°, but with a broader peak shape, suggesting that the layered sequence may be encapsulated. The background intensity is slightly raised in the 20–35° range, which can be attributed to the formation of amorphous gel C-S-H by cement hydration and secondary pozzolanic reaction.^73^^,^^74^ No low diffraction peaks appear near 18°, indicating that the Ca(OH)_2_ produced by cement hydration may be completely consumed by the secondary pozzolanic reaction of MK. This indicates that the cement-MK system generated additional amorphous binding gel, which filled pores, strengthened particle bonding, and reduced capillary connectivity. These mineralogical changes indicate increased formation of secondary cementitious gel and reduced capillary connectivity, which provides a direct microstructural basis for the lower permeability and improved freeze-thaw stability of CMS.

The XRD pattern of MGF-1.25% shows a significant increase in diffraction peak values near 14.005° and 16.853°, which may be due to increased local crystallinity caused by MGF-induced nucleation. This effect is consistent with nucleation-and-growth theory, where high-surface-energy interfaces increase the density of stable nuclei and improve the packing regularity of lamellar minerals or C-S-H clusters. This nucleation effect favors the formation of a denser fiber-matrix interphase, which helps restrain crack initiation and water ingress during freeze-thaw cycling and is consistent with the reduced permeability and improved residual mechanical performance of the MGF system.

The XRD pattern of RGF-1.25% shows weak absorption peaks at 27.916°, 36.436°, 39.882°, 55.124°, and 57.687°, while maintaining the original matrix minerals. These peaks indicate the successful deposition and retention of nano-TiO_2_ on the fiber surfaces and its involvement in matrix filling.^75^^,^^76^^,^^77^ The retention of the original mineral signatures indicates that TiO_2_ acts as a filler without altering the overall mineralogical framework. These observations suggest that TiO_2_ mainly acted as a micro/nano-filler and nucleation promoter rather than changing the bulk mineralogy. The resulting denser and more stable interphase likely reduced pore continuity, limited water migration, and suppressed freeze-thaw-induced crack coalescence, thereby providing a direct microstructural explanation for the lower permeability, reduced mass loss, and higher residual strength of the RGF system.

#### Fourier transform infrared (FTIR) analysis

**Figure 10:**
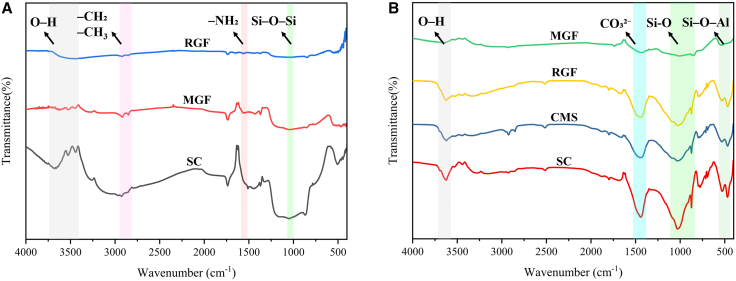
FTIR spectra identify the functional-group evolution associated with fiber modification and interfacial stabilization in the reinforced soil systems (A) FTIR spectra of different glass fibers. (B) FTIR spectra of different samples.

Figures 10A and 10B are Fourier transform infrared (FTIR) spectra. Figure 10A shows the spectra of GFs. The GF spectra show O–H stretching vibrations at 3443 cm^−1^ and 3531 cm^−1 79, 80^, indicating that the GF surface contains adsorbed water or surface -Si-OH hydroxyl groups.^78^^,^^79^ The FTIR spectrum exhibits a prominent Si-*O*-Si antisymmetric stretching vibration at 1000–1100 cm^−1 83^, confirming that the untreated GFs comprise an amorphous silica network. This network is primarily composed of Q^3^ and Q^2^ tetrahedral units with minor surface hydroxylation, resulting in low surface reactivity and high interfacial inertness.

The MGF spectrum shows that the absorption peaks of the hydroxyl group at 3531 cm^−1^ and 3443 cm^−1^ are smaller, and the absorption peak at 3678 cm^−1^ shifts to a lower frequency to 3613 cm^−1^ with a weakened peak value. This shift indicates that KH550 reacted with surface Si-OH groups to form Si-*O*-Si bonds, while the remaining hydroxyl groups became involved in stronger hydrogen bonding within the interfacial layer. In addition, a typical –NH_2_ bending vibration is observed at 1550 cm^−1 84^, which may be the result of KH550 grafting onto the fiber surface and retaining exposed amino groups. The absorption peaks at 2925 cm^−1^ and 2854 cm^−1^ are slightly enhanced, possibly due to the increase in silane organic chains, which further confirms the successful grafting of the KH550-containing cross-linked coating.^80^^,^^81^

The FTIR spectrum of RGF reveals a reconstruction of the hydrogen-bond network induced by the nano-TiO_2_ coating. The original hydroxyl peaks merge into a broad, unified band near 3531 cm^−1^, suggesting the formation of a multi-level hydrogen-bond network. Concurrently, the attenuation or disappearance of C-H and -NH_2_ peaks indicates that surface Ti–OH groups condense with silanols to form Ti-*O*-Si interfacial bonds, which partially bury or reorganize the silane-derived organic chains. These spectral features demonstrate a TiO_2_-driven vibrational mode homogenization and suppression of organic vibrational intensities, confirming the successful development of a more inorganic and structurally coherent interfacial layer around the fibers.

Figure 10B shows the FTIR spectra of different samples. The SC spectrum shows a significant inner-layer hydroxyl contraction at 3624 cm^−1^, exhibiting the characteristic inner-layer –OH band of kaolinite. These absorption bands indicate a kaolinite-Qtz dominated mineralogical framework, consistent with the layered phyllosilicate structure observed in XRD. Significant Si-O absorptions were observed at 1030 cm^−1^ and 780 cm^−1^, while distinct Si-*O*-Al absorption peaks were found at 530 cm^−1^ and 474 cm^−1^, indicating that the soil sample has a mineral structure dominated by kaolinite and Qtz.^82^

CMS spectra showed a significant broadening of the 1030 cm^−1^ region after the addition of cement and metakaolinite, corresponding to a background uplift in the 20–35° region of XRD. Both observations indicate the formation of a large amount of amorphous gel C-S-H. The absorption peak intensity at 3624 cm^−1^ decreased, indicating that hydration products progressively covered the clay surfaces and consumed reactive species. Together with the XRD results, these changes suggest the formation of a denser silicate/aluminosilicate gel network, which helps reduce pore connectivity and is therefore consistent with the improved strength and lower permeability of CMS.

The MGF sample spectrum shows that the absorption peaks at 3624 cm^−1^, 1442 cm^−1^, 1030 cm^−1^, and 780 cm^−1^ decrease or even disappear. This suggests that KH550 and the epoxy emulsion form a dense organic-inorganic hybrid layer on the GF surface, covering the functional groups of the particles surrounding it. This interfacial layer is beneficial for stabilizing the fiber-matrix bond and limiting interfacial debonding under freeze-thaw action.

The RGF sample spectrum shows that the absorption peaks at 530 cm^−1^ and 473 cm^−1^ are slightly enhanced, indicating that the hydration products such as Si-O and Al-O in the particles surrounding the RGF undergo local rearrangement, forming a more ordered structure, which is attributed to the TiO_2_-induced nucleation effect. The absorption peak at 1442 cm^−1^ is slightly enhanced because the high specific surface area of TiO_2_ absorbs CO_2_ and moisture from the air, generating carbonate species on its surface, which leads to the asymmetric stretching vibration of CO_3_^2^^,^^3^^,^^4^^,^^5^^,^^6^^,^^7^^,^^8^^,^^9^^,^^10^^,^^11^^,^^12^^,^^13^^,^^14^^,^^15^^,^^16^^,^^17^^,^^18^^,^^19^^,^^20^^,^^21^^,^^22^^,^^23^^,^^24^^,^^25^^,^^26^^,^^27^^,^^28^^,^^29^^,^^30^^,^^31^^,^^32^^,^^33^^,^^34^^,^^35^^,^^36^^,^^37^^,^^38^^,^^39^^,^^40^^,^^41^^,^^42^^,^^43^^,^^44^^,^^45^^,^^46^^,^^47^^,^^48^^,^^49^^,^^50^^,^^51^^,^^52^^,^^53^^,^^54^^,^^55^^,^^56^^,^^57^^,^^58^^,^^59^^,^^60^^,^^61^^,^^62^^,^^63^^,^^64^^,^^65^^,^^66^^,^^67^^,^^68^^,^^69^^,^^70^^,^^71^^,^^72^^,^^73^^,^^74^^,^^75^^,^^76^^,^^77^^,^^78^^,^^79^^,^^80^^,^^81^^,^^82^^,^^83^^,^^84^^,^^85^^,^^86^^,^^87^^,^^88^ These spectral changes indicate that nano-TiO_2_ promoted beneficial interfacial interactions and local structural ordering, in agreement with the XRD results. Overall, these FTIR features support the interfacial stabilization and gel-network densification discussed in Sections 2.4 and 2.5, rather than constituting an independent mechanistic explanation.

#### X-ray photoelectron spectroscopy (XPS) analysis

**Figure 11:**
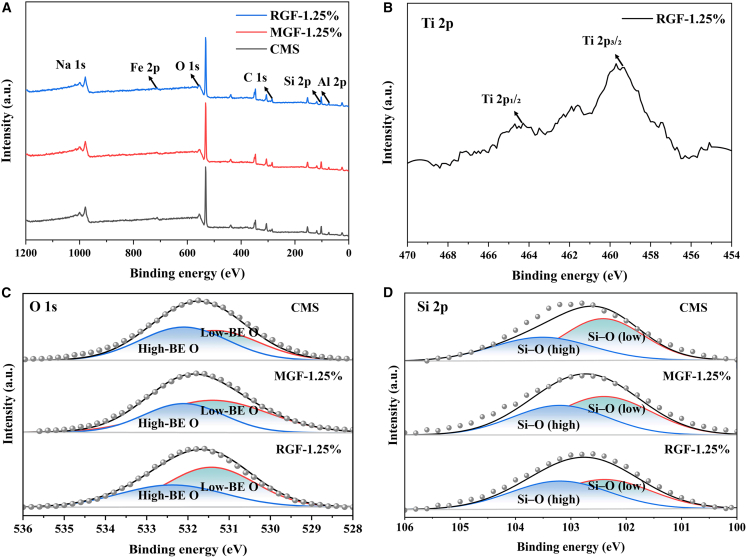
XPS characterization of CMS, MGF-1.25%, and RGF-1.25%: (A) survey spectra, (B) high-resolution Ti 2p spectrum of RGF-1.25%, (C) deconvoluted O 1s spectra, and (D) deconvoluted Si 2p spectra

To provide semi-quantitative evidence for the surface/interfacial chemical evolution associated with fiber functionalization and the interfacial strengthening mechanism proposed in Section 2.4, XPS was performed on CMS, MGF-1.25%, and RGF-1.25%. As shown in Figure 11A, all samples exhibit clear O 1s and Si 2p signals, whereas a distinct Ti 2p response appears only in RGF-1.25%, indicating the successful retention of Ti-containing species after fiber functionalization. The high-resolution Ti 2p spectrum of RGF-1.25% (Figure 11B) shows the characteristic Ti 2p3/2 and Ti 2p1/2 doublet, further supporting the introduction of nano-TiO_2_-related species into the interfacial region.

The deconvoluted O 1s spectra (Figures 11C; Table 3) can be resolved into a lower-binding-energy component at about 531.4 eV and a higher-binding-energy component at about 532.1–532.4 eV. Compared with CMS, the area fraction of the lower-binding-energy component increases from 40.74% to 54.38% in MGF-1.25% and further to 65.78% in RGF-1.25%, while the higher-binding-energy component correspondingly decreases from 59.26% to 45.62% and 34.22%, respectively. This shift suggests a more condensed and strongly bonded oxygen-containing interfacial environment after fiber modification. For MGF, this change is consistent with KH550-induced silane condensation and enhanced interfacial coupling. For RGF, the coexistence of the Ti 2p signal and the higher fraction of lower-binding-energy O 1s species suggests that nano-TiO_2_ participated in the reconstruction of the hybrid interfacial network and is consistent with the participation of Ti-containing species in interfacial chemical bonding.

**Table 3:** Deconvoluted XPS peak positions and area fractions of O 1s and Si 2p components for CMS, MGF-1.25%, and RGF-1.25%

Sample	O 1s Low-BE (eV)	O 1s Low-BE area (%)	O 1s High-BE (eV)	O 1s High-BE area (%)	Si 2p Low-BE (eV)	Si 2p Low-BE area (%)	Si 2p High-BE (eV)	Si 2p High-BE area (%)
CMS	531.4	40.74	532.1	59.26	102.4	59.09	103.5	40.91
MGF-1.25%	531.4	54.38	532.1	45.62	102.4	56.52	103.2	43.48
RGF-1.25%	531.4	65.78	532.4	34.22	102.4	50.00	103.2	50.00

Deconvoluted XPS peak positions and area fractions of O 1s and Si 2p components for CMS, MGF-1.25%, and RGF-1.25%. XPS, X-ray photoelectron spectroscopy; BE, binding energy. Low-BE and high-BE components represent the deconvoluted O 1s and Si 2p chemical environments used to compare the interfacial chemical evolution among CMS, MGF-1.25%, and RGF-1.25%.

The Si 2p spectra (Figures 11D; Table 3) show a similar tendency after fiber modification. The high-binding-energy Si component increases from 40.91% in CMS to 43.48% in MGF-1.25% and further to 50.00% in RGF-1.25%, whereas the low-binding-energy component decreases from 59.09% to 56.52% and 50.00%, respectively. This trend indicates an increased proportion of more polymerized siloxane/silicate environments after KH550 treatment and nano-TiO_2_ incorporation. In other words, the surface/interfacial chemical environment evolves from a relatively less condensed silicate/aluminosilicate environment in CMS to a more condensed hybrid structure in MGF and especially in RGF.

These XPS results provide semi-quantitative support for the interfacial strengthening mechanism proposed in Section 2.4, rather than serving as independent proof of all macroscopic property changes. The higher fraction of the lower-binding-energy O 1s component and the increased proportion of polymerized Si 2p environments indicate that KH550 promoted interfacial condensation, while nano-TiO_2_ further stabilized the interfacial structure through the participation of Ti-containing species and local chemical reorganization. This interpretation is consistent with the FTIR results in Section 2.6.2 and the MIP results in Section 2.5.3. Taken together, these findings help explain why the modified-fiber specimens exhibited superior compressive strength, shear strength, impermeability, and freeze-thaw resistance through the combined effects of physical fiber bridging and a chemically strengthened, structurally denser interphase.

#### Scanning electron microscopy (SEM) analysis

**Figure 12:**
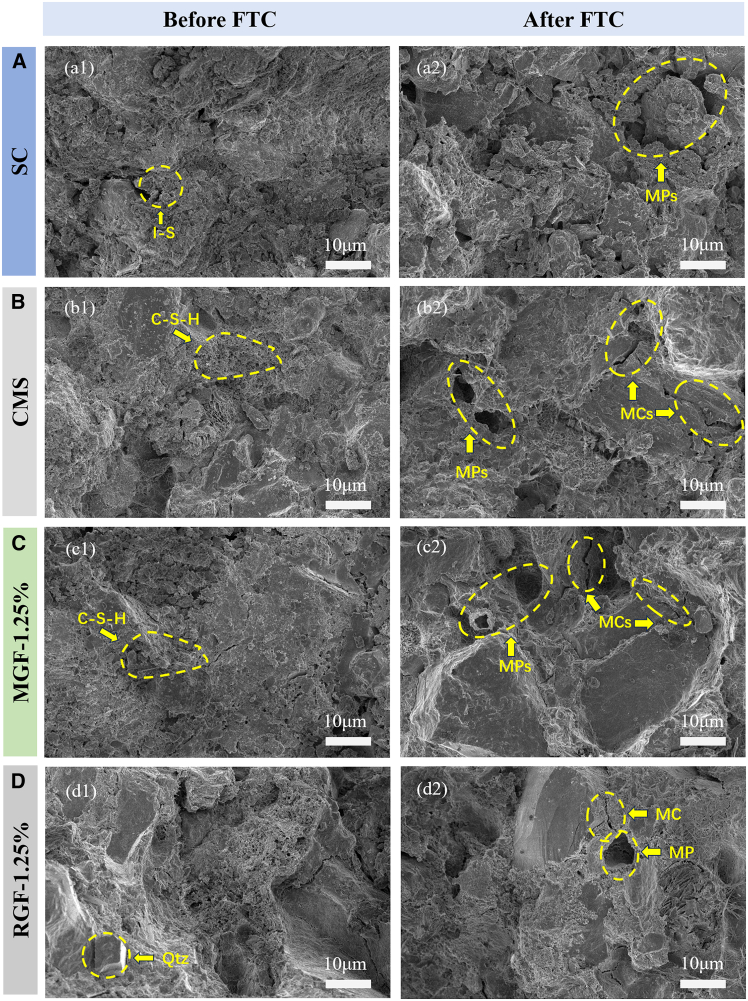
SEM test results of different materials before and after freeze-thaw cycling: (A) SC, (B) CMS, (C) MGF-1.25%, and (D) RGF-1.25%. Scale bars, 10 μm

Figure 12 shows the scanning electron microscopy (SEM) morphologies of the samples before and after FTC. Figure 12a1 shows the state of the SC before FTC, and Figure 12a2 shows the state of the SC after FTCs. Before FTC, SC shows a loose matrix of clay platelets and illite-smectite (I-S) mixed-layer minerals with limited cementation and visible micropores. After the FTC, micropores and early microcracks form due to hydraulic and crystallization pressures from the water-ice transition. Repeated expansion and contraction push particles apart and drive the growth and connection of these cracks. The coarsened pores increase flow-path continuity and rapidly reduce impermeability and mechanical stability.

Figure 12b1 shows the state before the CMS FTC, and Figure 12b2 shows the state after the CMS FTC. Before FTC, CMS contains abundant C-S-H gels and ettringite (AFt) crystals that fill voids and strengthen interparticle bonding. After the FTC, parts of this network break down, and micropores and microcracks appear around weakened gel bridges. Although the cemented structure delays damage compared with SC, ice-induced tensile stress fractures the brittle hydration products, reducing bonding and gel continuity. With more cycles, microcracks connect into branching networks, lowering load-transfer capacity, and frost resistance.

Figure 12c1 shows the state before the MGF-1.25% FTC, and Figure 12c2 shows the state after the MGF FTCs. Before FTC, MGF-1.25% shows dense hydration products attached to the functional coating (FC), forming a well-bonded fiber-matrix interface. After the FTC, only small and isolated micropores and microcracks appear. The coating improves mechanical interlocking and provides local stress buffering during ice expansion. The fibers bridge cracks and increase fracture-path tortuosity, limiting crack growth. These features are consistent with the better freeze-thaw stability of MGF than CMS observed in Section 2.5.

Figure 12d1 shows the state before the RGF-1.25% FTC, and Figure 12d2 shows the state after the RGF-1.25% FTCs. Before FTC, RGF-1.25% shows a rough, nano-TiO_2_-modified surface that creates a dense ITZ and promotes early hydration-product nucleation. After the FTC, only isolated microcracks appear, and no continuous cracks form. The rough surface enhances friction and interlocking, while the dense ITZ dissipates crack-tip energy. RGF fibers bridge and deflect cracks more effectively than MGF-1.25%, increasing tortuosity and preventing crack coalescence. These observations support the superior freeze-thaw resistance and interfacial stability of RGF discussed in Section 2.5.

Overall, SC and CMS follow the typical freeze-thaw deterioration route, namely microcrack initiation, propagation, coalescence, and fracture-network formation. In contrast, MGF-1.25% and RGF-1.25% delay crack initiation, increase crack tortuosity, and suppress crack connectivity. These effects preserve gel continuity and slow structural deterioration, with RGF showing the best performance because of its denser ITZ and stronger crack-bridging effect.

**Figure 13:**
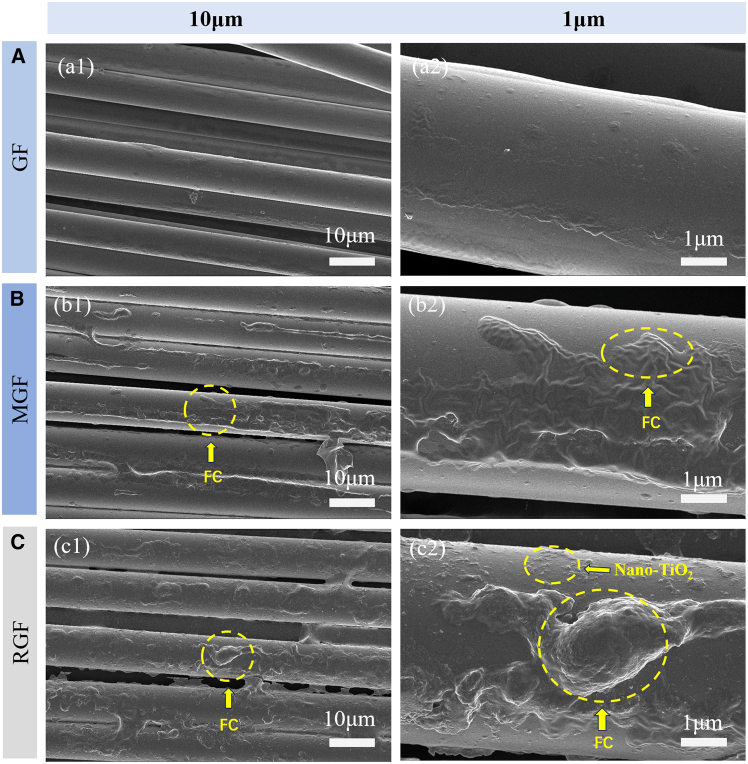
SEM test results of different glass fibers: (A) GF, (B) MGF, and (C) RGF. Scale bars, 10 μm in low-magnification panels and 1 μm in high-magnification panels

Figures 13A–13C show the SEM microstructures of different GFs. The SEM image of GF (Figure 13A) shows a relatively smooth surface with no obvious coating or deposits. The SEM image of MGF (Figure 13b1) at low magnification shows a uniformly distributed film layer covering the fiber surface, exhibiting a distinct convex condensation film; at high magnification (Figure 13b2), the continuous film layer formed by the crosslinking of epoxy emulsion, film-forming aids, and other components on the fiber surface displays a “wrinkled” or “wavy” FC. Compared to GF, the surface roughness is improved, indicating a clear film-forming system. The SEM image of RGF at low magnification (Figure 13c1) shows that multiple fiber surfaces are uniformly coated with a continuous and rough coating, with a locally connected network structure between the coatings of adjacent fibers, demonstrating that the modified system achieves effective and continuous coating on the fiber surface. Under high magnification (Figure 13c2), a distinct modified layer was observed on the fiber surface. This rough structure was synergistically constructed by aqueous nano-TiO_2_ and film-forming components: the dispersion of nano-TiO_2_ particles and the crosslinking of the film-forming agent jointly promoted the formation of protrusions and wrinkles.^89^ Compared with MGF, nanoparticle-induced discrete protrusions were observed, further enhancing the surface roughness.

All samples maintained continuous fiber geometry, indicating that the modification treatment did not damage the structural integrity of the fibers. In addition, both MGF and RGF exhibited distinct surface coatings, confirming the successful formation of a functional interfacial layer on the GF surface.

**Figure 14:**
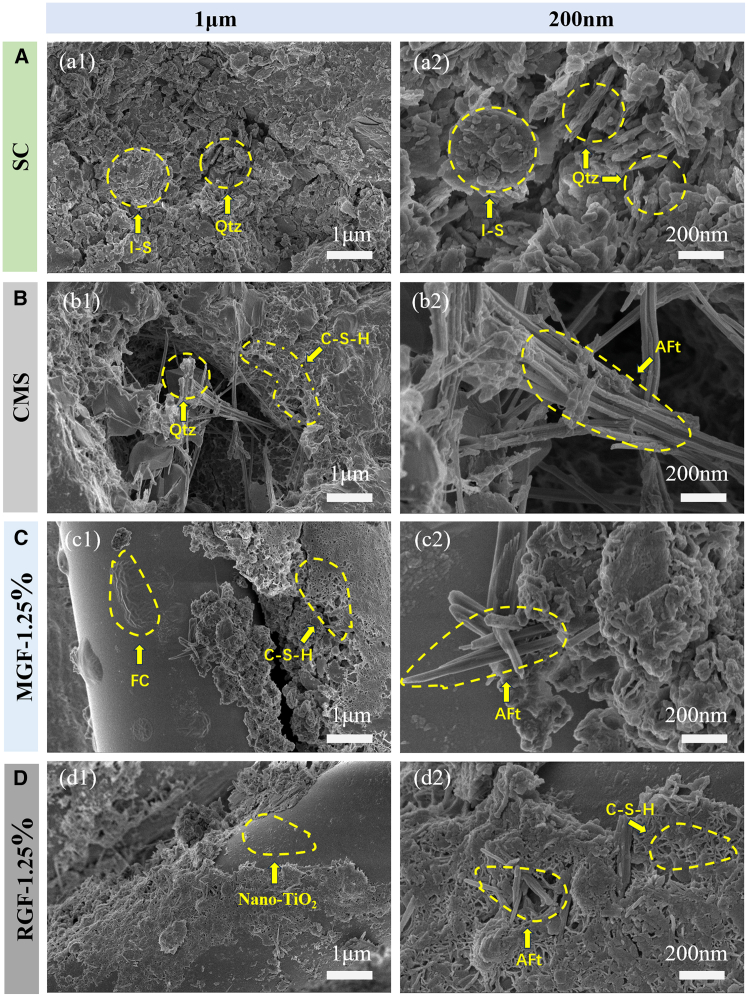
SEM test results of different stabilized silty clay systems: (A) SC, (B) CMS, (C) MGF-1.25%, and (D) RGF-1.25%. Scale bars, 1 μm in low-magnification panels and 200 nm in high-magnification panels

Figures 14A and 14B show the SEM microstructure characterization of different systems. Figure 14A shows the SEM image of SC. At low magnification, SC particles are densely packed, forming a relatively homogeneous but uncemented structure with no obvious exogenous phases. At high magnification, SC exhibits a typical clay particle aggregation morphology, with plate-like and flocculent structures stacked and aggregated. SEM images clearly reveal the characteristic platy-flaky morphology of illite-smectite mixed-layer minerals (I-S), displaying stacked lamellar aggregates with irregular edges. In contrast, Qtz appears as angular, blocky particles with smooth and sharp fracture surfaces, confirming its presence in the matrix. The particles are mainly bonded by van der Waals forces and electrostatic interactions,^90^^,^^91^ and numerous micropores exist at the interface.

Figure 14B shows the SEM image of CMS. At low magnification, the interfacial micropores are significantly reduced. At high magnification, the SEM microstructure displays bundles of slender, needle-like ettringite (AFt) crystals radiating from hydration sites, while the surrounding regions are filled with dense, foil-like and fibrous C-S-H gel, forming a continuous cementitious matrix. These products fill the gaps between clay particles, interweaving with them to construct a continuous cementitious network.^92^ Compared to SC, the pore volume is significantly reduced, and the overall structure exhibits stronger cohesion.

Figure 14C shows the SEM image of the MGF sample. Under low magnification, multiple MGF fibers are visible within the matrix, in addition to hydration products and clay particles. Cemented products fill the gaps between the fibers, indicating good adhesion between the MGF fibers and the matrix, demonstrating excellent fiber-soil interfacial bonding. Under high magnification, an organic modified layer on the MGF surface is clearly observed. This modified layer interacts with the surrounding cemented products, and the cemented products around the fibers are denser, indicating that the fibers guide the nucleation of the products. Compared with CMS, the overall structural continuity is improved, and the physical reinforcement mechanism is more pronounced.

Figure 14D shows the SEM image of the RGF sample. Under low magnification, obvious interweaving between fibers is visible, the fiber surface is rougher, the interfacial junction is uneven, and the frictional properties are more pronounced. Under high magnification, the interfacial bonding between the fibers and the matrix is exceptionally tight, with no obvious gaps. The surface exhibits a rough texture, the nanoparticles are uniformly distributed, and the chemical gel products are more densely distributed around the fibers, indicating a more pronounced induced nucleation effect. Compared to MGF, the microstructure exhibits superior continuity and uniformity.

### Conclusions

This study developed an interface-engineered glass-fiber-reinforced stabilized SC and evaluated its mechanical performance, permeability resistance, and freeze-thaw durability. A robust organic-inorganic hybrid coating was established via KH550 hydrolysis and epoxy compounding, while subsequent nano-TiO_2_ grafting yielded functionalized fibers with improved interfacial activity. Microscopic observations evidenced the formation of a dense, uniform coating, leading to superior bonding characteristics. The optimized composite exhibited notable improvements in mechanical strength (compressive strength of 4.98 MPa; *φ* = 38.14°, *c* = 555.45 kPa), permeability (3.02 × 10^−8^ cm/s), and freeze-thaw durability.

The hierarchical micro-nano interface effectively mitigated interfacial slippage, enabling stable load transfer and improved structural stability under cyclic environmental actions. The reduced permeability and enhanced freeze-thaw resilience indicate strong suitability for cold-region geotechnical applications, foundations in water-sensitive areas, and transport infrastructure subjected to dynamic loading. These results demonstrate that the proposed composite presents a promising, efficient stabilization solution with reduced dependency on cement-based approaches.

Mechanistically, the results highlight that a hybrid interface combining chemical bonding (KH550–epoxy), nano-TiO_2_ anchoring, and micro-scale mechanical interlocking can synergistically enhance reinforcement efficiency. The demonstrated linkage between fiber-scale interfacial tailoring and macro-scale geotechnical performance provides a foundation for the design of next-generation stabilization materials and micro/nano-functionalized geocomposites.

### Limitations of the study

This study demonstrates that interface-engineered GFs can improve the mechanical performance, permeability resistance, and freeze-thaw durability of cement-MK-stabilized SC. However, several limitations should be noted. First, the freeze-thaw tests were conducted under sealed moisture boundary conditions to minimize uncontrolled moisture exchange and isolate intrinsic thermal damage. This condition differs from field subgrade environments, where continuous water replenishment, drainage, seasonal moisture migration, and traffic-induced loading may accelerate deterioration. Second, the nano-TiO_2_ dosage used in the soil composite tests was selected based on the tensile performance of reinforced GFs. Therefore, the selected dosage should be regarded as an effective interfacial-enhancement condition rather than a globally optimized nano-TiO_2_ content for the soil composite system. Third, the present work was mainly conducted using laboratory-scale specimens, and field-scale validation under realistic stress states and environmental boundary conditions is still needed. Finally, although the cement-MK system may reduce cement dependence at the mix-design level, a complete life cycle assessment or direct CO_2_-emission quantification was not conducted.

## Resource availability

### Lead contact

Further information and requests for resources should be directed to and will be fulfilled by the lead contact, Zhengjun Wang (wangjiaoshou2025@163.com).

### Materials availability

This study generated MGF and RGF through the surface functionalization of commercial glass fibers using KH550, epoxy resin, and nano-TiO_2_. Additional information on material preparation and experimental protocols is available from the lead contact upon reasonable request.

### Data and code availability

• **Data:** The standardized dataset generated and analyzed in this study is publicly available in Figshare at https://doi.org/10.6084/m9.figshare.30759491. The dataset is fully public and is not subject to any embargo, delayed-release, or restricted-access arrangement. Additional information is available from the lead contact upon reasonable request.
• **Code:** This study did not generate or use any custom code.
• **Other:** No additional resources are associated with this study.

## Acknowledgments

This work was supported by the Heilongjiang Provincial Key R&D Program Guidance Category Scientific Research Projects (GZ20220138), the Scientific Research Program of the Department of Ecology, and the Environment of Heilongjiang Province (HST2022GF004).

## Author contributions

Conceptualization, B.P. and Z.W.; methodology, B.P. and Z.W.; software, B.P.; validation, B.P.; formal analysis, B.P.; investigation, B.P.; resources, Z.W. and X.Y.; data curation, B.P.; writing – original draft preparation, B.P.; writing—review and editing, B.P.; visualization, B.P.; supervision, Z.W., X.Y., and R.Z.; project administration, Z.W. and B.P.; funding acquisition, Z.W. and X.Y. All authors have read and agreed to the published version of the manuscript.

## Declaration of interests

The authors declare no competing interests.

## STAR★Methods

### Key resources table

**Table:** 

REAGENT or RESOURCE	SOURCE	IDENTIFIER
**Chemicals, peptides, and recombinant proteins**		

Silty clay	Linyi County, Shanxi, China	N/A
Ordinary Portland cement P·O 42.5	Liaoning Yinsheng Cement Group	N/A
Metakaolin	Henan Borun Foundry Materials	N/A
Glass fiber (GF)	Changzhou Hongtu Composite Materials Co., Ltd., China	N/A
KH550 (γ-aminopropyltriethoxysilane)	Shanghai Maclean Biochemical	N/A
Nano-TiO_2_ aqueous dispersion	Shanghai Yingcheng New Materials	30 wt % rutile
Epoxy emulsion (EE)	Shanghai Lvjia Waterborne Coatings Co., Ltd.	Series GEM
PVA film-forming aid	Arles New Materials	N/A

**Deposited data**		

Experimental datasets generated in this study	This paper	Figshare: https://doi.org/10.6084/m9.figshare.30759491

**Software and algorithms**		

Origin	OriginLab	https://www.originlab.com
Microsoft Excel	Microsoft	https://www.microsoft.com/microsoft-365/excel

**Other**		

Photoelectric liquid–plastic limit tester	Suzhou Institute of Testing Technology Application	SJS-3
Universal testing machine (UCS)	Jinan Shidai Shijin	WDW-100 E
Direct shear apparatus (DST)	Cangzhou Yixuan Test Instrument Co	ZL
Variable-head soil permeameter	Xianxian Keyu High-speed Railway Instrument Equipment Factory	TST-55
X-ray diffractometer	Rigaku	D-MAX 2500/PC
FTIR spectrometer	Thermo Scientific	iS50
Mercury intrusion porosimeter	Micromeritics	AutoPore V 9600
XPS spectrometer	Thermo Scientific	ESCALAB 250Xi
Scanning electron microscope	Carl Zeiss	Sigma 500

### Method details

#### Materials

This study used the following materials. The base soil was silty clay (SC) from Linyi County, Shanxi, China (110°17′30.7″E, 34°58′52.9″N). The cementitious system consisted of ordinary Portland cement (P·O 42.5, Liaoning Yinsheng Cement Group) as the primary binder and metakaolin (Henan Borun Foundry Materials) for microstructural refinement and durability enhancement, their chemical compositions are summarized in the table below.

For fiber functionalization, a silane coupling agent KH550 (C9H23NO3Si, Shanghai Maclean Biochemical) provided molecular bonding sites, while an aqueous nano-TiO_2_ dispersion (30% rutile, Shanghai Yingcheng New Materials) enabled the construction of an inorganic hydrogen-bond network. Processing aids included dioleoyl triglyceride (filament lubricant) and a polyvinyl alcohol (PVA) film-forming aid (Arles New Materials). Purified water and analytical-grade reagents (anhydrous ethanol and glacial acetic acid) were used throughout. Material morphologies are presented in Figure 15.

**Table:** Chemical composition of cement and metakaolin

Material	Al_2_O_3_ (%)	SiO_2_ (%)	Fe_2_O_3_ (%)	CaO (%)	MgO (%)	SO_3_ (%)	LoI (%)	Other substances (%)
Cement	8.26	24.99	4.03	51.42	3.71	2.51	3.31	1.77
Metakaolin	45.58	51.58	0.59	–	–	–	0.11	0.88

**Figure:**
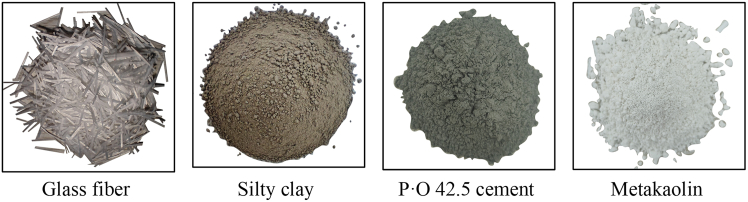
Experimental materials

#### Test plan

##### Determination of basic properties of test soil samples

The plasticity index of the soil was determined with reference to GB/T 50123–2019. The soil was first oven-dried at 105 °C and sieved through a 0.5 mm mesh to obtain a uniform sample. Three 200 g portions were then prepared at distinct moisture states, corresponding to a crumb condition, a non-crumbling state, and a denser plastic state, as detailed in the combined liquid–plastic limit mix-proportion table below. After thorough mixing and 24 h of sealed equilibration at 24 °C to ensure moisture equilibrium, the cone penetration depth and moisture content of each sample were measured using a combined liquid-plastic limit apparatus. The plasticity index was subsequently derived from the fitted relationship between penetration depth and moisture content.

**Table:** Standard specimen mix proportions for combined liquid–plastic limits test

No.	SC(g)	W(g)
LP-1	200	48.4
LP-2	200	36.6
LP-3	200	24.6

A series of soil mixtures with controlled moisture contents (480–800 g of water per ∼4 kg soil) were prepared from a 20 kg bulk sample by quartering, with the mix proportions listed in the SC compaction mix-proportion table below. After 12 h of sealed equilibration at 24 °C to ensure moisture equilibrium, samples were compacted in accordance with GB/T 50123–2019. The wet density, derived from the sample mass (*m*_0_) and ring volume, and the gravimetric moisture content (*w*) were used to calculate the dry density. The optimum moisture content and corresponding maximum dry density were then obtained from the peak of the least-squares-fitted dry density–moisture content curve.

**Table:** Standard mix proportions for compaction test specimens (SC)

No.	SC(g)	W(g)
SC-1	4000	480
SC-2	4000	560
SC-3	4000	640
SC-4	4000	720
SC-5	4000	800

##### Preparation of modified and enhanced glass fibers

A controlled hydrolysis–coating procedure was used to prepare the modified glass fibers. First, anhydrous ethanol (EtOH) and deionized water (W) were mixed at a mass ratio of 3:7, and the pH was adjusted to 5.0 with glacial acetic acid (GAA) to create a suitable hydrolysis environment. The solution was maintained at 25 °C in a constant-temperature bath to avoid premature condensation, after which KH550 (0.5 wt %) was introduced and hydrolyzed under stirring for 30 min. The temperature was then increased to 60 °C, and film-forming aid (FiFA, 0.3 wt %), epoxy emulsion (EE, 3.0 wt %), and dioleoyl triglyceride (PG-3·DOT, 0.4 wt %) were added sequentially. Continued stirring for 60 min facilitated crosslinking and the formation of a transient physical network, yielding a stable coating system. For MGF preparation, glass fibers were immersed in the coating for 10 min and subsequently dried at 110 °C for 10 min.

Based on this process, nano-TiO_2_ (0.25–1.50 wt %) was incorporated during the coating-formation stage to obtain reinforced glass fibers (RGF). After impregnation and drying, the nano-TiO_2_ particles were uniformly anchored onto the functionalized fiber surface. The workflow, reaction scheme, and reinforcement mechanism are presented in Figures 16A–16C. Because epoxy emulsions contain high-molecular-weight polymers with complex structures, the mechanistic description adopts a simplified epoxy model molecule (C_3_H_5_ClO) to represent the epoxy component. To determine the optimal nano-TiO_2_ dosage, tensile strength tests were performed on all RGF formulations, as summarized in the glass-fiber formulation table below.

**Table:** Mix proportions for specimens with different glass fiber types

No.	W(g)	EtOH(g)	FiFA(g)	EE(g)	PG-3·DOT(g)	Nano-TiO_2_(g)
MGF	560	240	2.4	24	3.2	0
RGF-0.25	560	240	2.4	24	3.2	2
RGF-0.50	560	240	2.4	24	3.2	4
RGF-0.75	560	240	2.4	24	3.2	6
RGF-1.00	560	240	2.4	24	3.2	8
RGF-1.25	560	240	2.4	24	3.2	10
RGF-1.50	560	240	2.4	24	3.2	12

**Figure:**
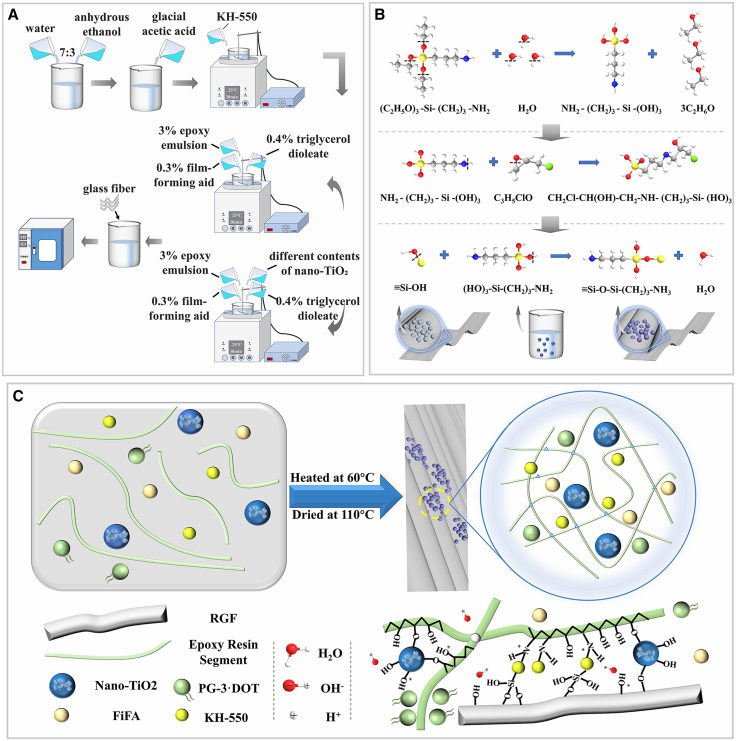
The proposed modification and reinforcement mechanisms illustrate how hierarchical interfacial engineering improves fiber-matrix bonding (A) Glass fiber modification process. (B) Scheme of chemical reaction principle. (C) Schematic diagram of the bridging mechanism.

##### Determination of the pptimum mix proportions of cement and metakaolin

An orthogonal experimental design was used to preliminarily screen cement–metakaolin combinations based on early-age shear performance. The selected dosage ranges were informed by published studies on SC stabilization using metakaolin- or geopolymer-based binders, which indicate that low-to-moderate binder additions can effectively improve soil performance.^93^ Considering that cement served as the primary binder and metakaolin as a supplementary pozzolanic material in the present system, cement contents of 4%, 6%, and 8% and metakaolin contents of 2%, 3%, and 4% were selected for preliminary orthogonal screening, as listed in the CMS orthogonal mix-design table below. Specimens were prepared using a ring cutter (61.8 mm diameter, 20 mm height) and cured for seven days. Direct shear tests were then conducted under a normal stress of 15.01 kPa to evaluate shear performance.

**Table:** Orthogonal mix design of CMS specimens

No.	SC(g)	C(g)	MK(g)	W(g)
CMS-4-2	160	6.4	3.2	25.95
CMS-4-3	160	6.4	4.8	26.19
CMS-4-4	160	6.4	6.4	26.44
CMS-6-2	160	9.6	3.2	26.44
CMS-6-3	160	9.6	4.8	26.68
CMS-6-4	160	9.6	6.4	26.93
CMS-8-2	160	12.8	3.2	26.93
CMS-8-3	160	12.8	4.8	27.17
CMS-8-4	160	12.8	6.4	27.42

CMS readily absorbs moisture through physical adsorption and hydration, so a compaction test was conducted to determine the optimal moisture content for subsequent specimen preparation. The corresponding mix proportions are presented in the CMS compaction mix-proportion table below.

**Table:** Standard mix proportions for compaction test specimens (CMS)

No.	SC(g)	C(g)	MK(g)	W(g)
CMS-1	4000	320	120	621.6
CMS-2	4000	320	120	710.4
CMS-3	4000	320	120	799.2
CMS-4	4000	320	120	888
CMS-5	4000	320	120	976.8

##### Test soil sample performance testing

SC and CMS were prepared following their optimized mix designs. The soil, cement, metakaolin, water, and fibers were mixed in a controlled sequence to ensure uniform distribution. Composite soil specimens were prepared by incorporating MGF or RGF at mass fractions of 0.25%–1.50% into the cement–metakaolin matrix. The dry materials were premixed for 3–5 min, followed by gradual water addition with continued mixing for 4–6 min. The mixture was compacted into cylindrical specimens using standard compaction procedures. Three parallel specimens (*n* = 3) were prepared for each group. The specimen preparation procedure is shown in Figure 17. After curing, unconfined compressive strength, direct shear, impermeability, and freeze–thaw tests were performed. The specific mix proportions used for each test group are summarized in the CMS compaction mix-proportion table below.

**Table:** Standard sample formulation

No.	SC(g)	MK(g)	C(g)	GF(g)	W(g)
SC	230	0	0	0	35.19
CMS	230	6.9	18.4	0	46.21
MGF-0.25	230	6.9	18.4	0.575	46.21
MGF-0.5	230	6.9	18.4	1.15	46.21
MGF-0.75	230	6.9	18.4	1.725	46.21
MGF-1	230	6.9	18.4	2.3	46.21
MGF-1.25	230	6.9	18.4	2.875	46.21
MGF-1.5	230	6.9	18.4	3.45	46.21
RGF-0.25	230	6.9	18.4	0.575	46.21
RGF-0.5	230	6.9	18.4	1.15	46.21
RGF-0.75	230	6.9	18.4	1.725	46.21
RGF-1	230	6.9	18.4	2.3	46.21
RGF-1.25	230	6.9	18.4	2.875	46.21
RGF-1.5	230	6.9	18.4	3.45	46.21

**Figure:**
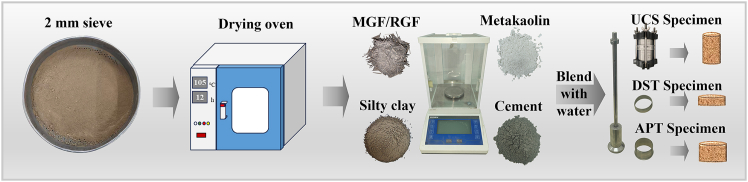
Test soil block preparation process

#### Test methods

##### Combined determination of liquid and plastic limits

The liquid limit and plastic limit tests were conducted in accordance with GB/T 50123–2019. The plasticity index was determined using a photoelectric liquid–plastic limit tester (SJS-3, Suzhou Institute of Testing Technology Application) equipped with a 100 g, 30° cone. The sample cup was lightly lubricated, and soil at the target moisture content was placed in three layers with minimal disturbance. After leveling the surface, the cone was released to penetrate the soil, and the penetration depth (*h*) was recorded. A 20 g subsample was collected for moisture content determination using the oven-drying method, according to Equation 1. A double-logarithmic plot of moisture content (*w*) versus penetration depth (*h*) was constructed, and the liquid and plastic limits were obtained from the fitted curve. The plasticity index was then calculated according to Equation 2.

(Equation 1) $$w=\left(\frac{m_{0}}{m_{d}}-1\right)\times 100\%$$

where *w* is the moisture content (%); *m*_0_ is the wet soil mass (g); *m*_d_ is the dry soil mass (g).

(Equation 2) IP=wL−wP

where I*p* is the plasticity index (%); *w*_L_ is the liquid limit (%); *w*_P_ is the plastic limit (%).

##### Compaction test

The compaction test was conducted in accordance with GB/T 50123–2019. The compaction behavior was evaluated using a lightweight compactor (mold diameter 150 mm, height 116 mm; hammer mass 2.5 kg; drop height 305 mm). A thin layer of lubricant was applied to the mold interior, and the soil at the target moisture content was compacted in three layers, with 25 blows per layer. The compacted specimen was extruded and trimmed using a ring cutter. Its mass (*m*_0_) was recorded, and a 20 g subsample was oven-dried to determine the moisture content (*w*). The dry density was calculated using Equation 3. Dry density was plotted against moisture content, and a quadratic fit obtained by least-squares regression was used to determine the maximum dry density and the corresponding optimum moisture content.

(Equation 3) $$\rho =\frac{m_{0}}{v}$$

where *ρ* is the wet density (g/cm3); *v* is the ring cutter (cm3); *m*_0_ is the ring cutter cutting quality (g).

(Equation 4) $$\rho_{d}=\frac{\rho }{1+0.01w}$$

where *ρ*_d_ is the dry density (g/cm3); *ρ* is the wet density (g/cm3); *w* is the moisture content (%).

##### Glass fiber tensile strength test

According to GB/T 7690.3–2013, the tensile performance of the fibers was evaluated using a displacement-controlled loading protocol. A gauge length of 350 mm and an axial loading rate of 200 mm/min were adopted to ensure consistency across specimens. Ten replicates were tested for each fiber category, and the breaking strength was determined from the peak load recorded during testing.

##### Unconfined compression test

The unconfined compression test was conducted in accordance with GB/T 50123–2019. The unconfined compressive strength of the composite soil was evaluated under monotonic axial loading using a displacement-controlled universal testing system. Standard cylindrical specimens (39.1 mm × 80 mm) were loaded at 1 mm/min until peak resistance, while real-time load–displacement data were recorded for UCS determination (Equation 5).

(Equation 5) $$\sigma_{S}=\frac{P}{A}$$

where *σ*_S_ is the unconfined compressive strength of the specimen (kN/m^2^); *P* is the maximum axial load applied to the specimen (kN); *A* is the cross-sectional area of the specimen (m^2^).

##### Direct shear test

The direct shear test was conducted in accordance with GB/T 50123–2019. The shear behavior of the composite soil was evaluated using a consolidation–rapid shear protocol with a strain-controlled direct shear apparatus. Cylindrical specimens (61.8 mm × 20 mm) were placed between permeable stones with filter papers to ensure uniform drainage. After applying the designated normal stresses (5, 10, 15, and 20 kPa), shearing was conducted at a constant displacement rate of 0.8 mm/min until peak resistance was reached. The corresponding peak shear stresses were determined using Equation 6. By plotting shear stress versus normal stress, the cohesion (*c*) and internal friction angle (*φ*) were obtained from the intercept and slope of the linear failure envelope, respectively.

(Equation 6) $$\tau =\frac{C\times R}{A_{0}}\times 10$$

where *τ* is the shear stress (kPa); *C* is the dynamometer calibration coefficient (N/0.01 mm); *R* is the dynamometer readings (0.01 mm); *A*_0_ is specimen cross-sectional area (cm^2^).

##### Permeation test

The permeability test was conducted in accordance with GB/T 50123–2019. Permeability was evaluated using a variable-head method in accordance with standard procedures. Cylindrical specimens (61.8 mm × 40 mm) were pre-saturated for 24 h to ensure full water uptake before testing. During the test, the specimen was vertically confined in the permeation cell, and the head difference was monitored continuously after stabilization. The initial and final water head heights (*h*_1_ and *h*_2_) and the corresponding elapsed time (t) were recorded, and the permeability coefficient was calculated using Equation 7. This protocol ensures reliable quantification of the hydraulic response of stabilized SC under low-permeability conditions.

(Equation 7) $$k=\frac{a\times L}{A\times t}\times \ln\left(\frac{h_{1}}{h_{2}}\right)$$

where *k* is the permeability coefficient (cm/s); *a* is the cross-sectional area of the variable head pipe (cm^2^); *A* is the cross-sectional area (cm^2^); *L* is the sample length (cm); *h*_1_, *h*_2_ are the initial and head heights (cm); *t* is the time taken for head change (s).

##### Freeze-thaw cycle test

After 28 days of standard curing, the specimens were surface-dried, sealed with plastic film to prevent moisture loss, and subjected to unidirectional freeze–thaw cycling in a customized chamber. Each freeze–thaw cycle consisted of 12 h at −25 °C and 12 h at 25 °C. Specimens were tested after 5, 10, 15, and 20 cycles. Before testing, specimens were equilibrated to room temperature. Three parallel specimens (*n* = 3) were tested for each condition. At each interval, mass, unconfined compressive strength, and impermeability were measured, and the corresponding mass-loss rate and residual strength were calculated using Equations 8 and 9. The results were used to evaluate the degradation behavior and durability evolution under repeated F–T loading. It should be noted that the specimens were sealed during freeze–thaw cycling to minimize uncontrolled moisture exchange and isolate intrinsic thermal damage effects. Consequently, the present protocol mainly represents a closed-moisture boundary condition rather than a field condition with continuous external water replenishment. Under field conditions involving continuous water replenishment, the deterioration rate may be more severe.

(Equation 8) $$W_{n}=\frac{m_{0}-m_{n}}{m_{0}}\times 100\%$$

where *W*_*n*_ is the mass loss rate after n FTCs (%); *m*_0_ is the sample mass before FTC (g); *m*_*n*_ is the mass after n FTCs (g).

(Equation 9) $$Rn=\frac{f_{n}}{f_{0}}$$

where *f*_0_ is the unconfined compressive strength before FTC (MPa); *f*_*n*_ is the absolute residual strength of the sample after n FTCs (MPa); *R*_*n*_ is the relative residual strength.

##### Characterization

X-ray diffraction (XRD) patterns were acquired on a Rigaku D-MAX 2500/PC instrument (Cu Kα, λ = 1.5418 Å) over a 2θ range of 5–85° with a 0.02° step size.

Fourier transform infrared (FTIR) spectroscopy was performed using a Thermo Scientific iS50 spectrometer equipped with a diamond attenuated total reflectance (ATR) accessory. Spectra of the glass fibers and bulk samples were acquired in ATR mode over a range of 4000–400 cm^−1^ at a resolution of 4 cm^−1^ with 32 scans per measurement.

Mercury intrusion porosimetry (MIP) was performed using a Micromeritics AutoPore V 9600 instrument over a pressure range of 0.10–61,000 psia. CMS, MGF-1.25%, and RGF-1.25% specimens after 20 freeze–thaw cycles were analyzed to obtain the total intrusion volume, porosity, median pore diameter (volume), and average pore diameter (4 V/A).

X-ray photoelectron spectroscopy (XPS) was performed using an ESCALAB 250Xi spectrometer with a monochromatic Al Kα source (hν = 1486.6 eV). The binding energies were calibrated against the C 1s peak at 284.8 eV, and the spectra were fitted after Shirley background subtraction.

Scanning electron microscopy (SEM) was performed on a Carl Zeiss Sigma 500 instrument at 3 kV on samples that were pre-dried (60 °C, 6 h) and sputter-coated with ∼5 nm of gold to ensure conductivity.

### Quantification and statistical analysis

Quantitative data are presented as mean ± standard deviation (SD) unless otherwise stated. For liquid–plastic limit, compaction, unconfined compressive strength, direct shear, permeability, and freeze–thaw durability tests, *n* = 3 independent specimens or measurements were used for each group. For the glass fiber tensile test, *n* = 10 fibers were tested for each formulation. Here, n represents the number of independent specimens or measurements.

Error bars in the figures represent SD around the mean. Statistical details, including the exact value of n and error-bar definitions, are provided in the corresponding figure legends and, where appropriate, in the Results section. No asterisk annotations are used in the figures.

A two-way analysis of variance (ANOVA) was used to evaluate the effects of cement content, metakaolin content, and their interaction on the shear strength of cement–metakaolin-stabilized silty clay in the orthogonal experiment. A *p* value <0.05 was considered statistically significant where statistical testing was performed. Least-squares regression was used to fit the liquid–plastic limit relationship and the compaction curve for determining the plasticity parameters, optimum moisture content, and maximum dry density. Data processing and plotting were performed using Microsoft Excel and Origin.

### Additional resources

This study did not generate additional resources.

## References

[1] Hu J., Zhou B., Li P., Wang J., Yang Y. (2025). Pressure-Amplified Structural Superiority in Silty Clays: Dynamic Divergence Between Undisturbed and Remolded States. Buildings.

[2] Zhang Z.L., Cui Z.D., Xu M.Z. (2025). Experimental investigation of the undrained dynamic behavior of soft clay under equivalent earthquake loadings. Soil Dynam. Earthq. Eng..

[3] Wang A., Zhang C., Luo T., Ma C., Zhao Y., You Z., Sun X., Li Y., Song Y., Yang W. (2025). Mechanical properties of consolidated water-saturated natural gas hydrate-bearing silty-clayey sediments under undrained shearing conditions. Int. J. Hydrogen Energy.

[4] Shi Y., Li S., Zhang T., Liu J., Zhang J. (2024). Compaction and shear performance of lime-modified high moisture content silty clay. Case Stud. Constr. Mater..

[5] Sett S., Chattopadhyay K.K., Ghosh A. (2024). Reliability-based seismic liquefaction hazard mapping of Kolkata Metropolitan City, India, using ordinary kriging technique. Bull. Eng. Geol. Environ..

[6] Liu Q.q., Xie W.l., Yang H., Yan M., Zhu R.s., Mu K., Chang Y.l., Di S., He X., Liu X., Zhang Y. (2025). Characteristics of loess landslides under the effects of faults and rivers and their correlations with geological and surficial evolutionary processes. Bull. Eng. Geol. Environ..

[7] Liu Q., Xie W., Yang H., Yuan K., Zhang S., Li X., Qu P., Wu Z., Zhou J., Gao X. (2025). Intrinsic Mechanisms of Differences in Wetting-Induced Deformation of Soils on Chinese Loess Plateau: Insights into Land Stability and Sustainable Management. Land.

[8] Tang Q., Sun C., Chen Y., Guo W., Jia R., Gao H., Zhou Y., Chen W., Han G., Xu X. (2024). Impact of clay on the decompositional mechanical properties of clayey silt hydrate sediments. Energy Fuels.

[9] Bu F., Liu J., Mei H., Song Z., Wang Z., Dai C., Qian W. (2023). Cracking behavior of sisal fiber-reinforced clayey soil under wetting-drying cycles. Soil Tillage Res..

[10] Hu C., Tian Y., Liu X., Jia Y. (2024). Permeability of surface clay-bearing sediments in Shenhu Area of South China Sea. Eng. Geol..

[11] Wu H., Shao S., Shao S., Wang Z., Yan G. (2024). Cyclic loading deformation response and microstructure evolution of undisturbed loess subjected to pre-humidification process. Eng. Geol..

[12] Deng F., Lu J., Wan X., Liu B., Zhang B., Fu H. (2025). Mitigating frost heave of a soil stabilized with sisal fiber exposed to freeze-thaw cycles. Geotext. Geomembranes.

[13] Almeida M., Marques M., Riccio M., Fagundes D., Lima B., Polido U., Cirone A., Hosseinpour I. (2022). Ground improvement techniques applied to very soft clays: state of knowledge and recent advances. S&R..

[14] Wang X., Cheng C., Li J., Zhang J., Ma G., Jin J. (2023). Automated monitoring and evaluation of highway subgrade compaction quality using artificial neural networks. Autom. ConStruct..

[15] Niu W., Guo B., Li K., Ren Z., Zheng Y., Liu J., Lin H., Men X. (2024). Cementitous material based stabilization of soft soils by stabilizer: Feasibility and durabiliy assessment. Constr. Build. Mater..

[16] Gao Q., Ge J., Zhang J., Ren Z., Wu D., Cheng G., Zhang K. (2023). Experimental study on the engineering characteristics of modified silt in the Yellow River alluvial plain. Constr. Build. Mater..

[17] Zhang X., Liu Z., Han Y. (2024). Progress towards the identification and improvement of dispersive soils: A review. Eur. J. Soil Sci..

[18] Zhou Y., Huo M., Zhang L., Guan Q. (2024). Strength development and solidification mechanism of soils with different properties stabilized by cement-slag-based materials. Case Stud. Constr. Mater..

[19] Ge W., Zhang Z., Ashour A., Li W., Jiang H., Hu Y., Shuai H., Sun C., Li S., Liu Y., Cao D. (2023). Hydration characteristics, hydration products and microstructure of reactive powder concrete. J. Build. Eng..

[20] Rasool Haji T., Ahmed Mir B. (2023). Effect of nano-gypsum on mechanical properties cement admixed marginal silty soil. Constr. Build. Mater..

[21] Xu J., Guan Y., Oldfield J., Guan D., Shan Y. (2024). China carbon emission accounts 2020-2021. Appl. Energy.

[22] Wu S., Shao Z., Andrew R.M., Bing L., Wang J., Niu L., Liu Z., Xi F. (2024). Global CO2 uptake by cement materials accounts 1930–2023. Sci. Data.

[23] Chen H., Li N., Unluer C., Chen P., Zhang Z. (2025). 200 years of Portland cement: Technological advancements and sustainability challenges. J. Clean. Prod..

[24] Pobłocki K., Pawlak M., Drzeżdżon J., Gawdzik B., Jacewicz D. (2024). Clean production of geopolymers as an opportunity for sustainable development of the construction industry. Sci. Total Environ..

[25] Madirisha M.M., Dada O.R., Ikotun B.D. (2024). Chemical fundamentals of geopolymers in sustainable construction. Mater. Today Sustain..

[26] Liao G., Wang D., Wang W., He Y. (2024). Microstructure, strength development mechanism, and CO2 emission analyses of alkali-activated fly ash-slag mortars. J. Clean. Prod..

[27] Gong M., Shen A., Wang Y., Lin H., He R. (2025). Alkali and sulfate effects on mechanical properties and microscopic mechanisms of slag and fly ash geopolymers. Sci. Rep..

[28] Li L., Zheng X., Wu J., Zhang J., Li P., Wei X. (2024). Performance of the one-part geopolymer stabilized soft clay under acids attack. J. Clean. Prod..

[29] Lin H., Sui Z., Li F., Wu W. (2025). Materials-empowered smart versatile building envelopes toward a sustainable energy-water-environment nexus. Matter.

[30] Dong W., Ma M. (2025). Recent developments and advanced applications of promising functional nanocomposites for green buildings: A review. J. Build. Eng..

[31] Zhou Y., Zhong X., Zhao C., Luo T., Tian K., Li Y. (2025). Study on the strengthening and toughening mechanism of ultra-high-performance concrete through hydrogen peroxide activation and corrosion of steel fibers with surface-anchored carbon nanotubes. Constr. Build. Mater..

[32] Vinodh D., Lakshmaiya N., Kumar T.R.S., Kaliappan S., Balaji V., Ross N.S., Maranan R. (2025). Sustainable hybrid biocomposites using agricultural waste fillers and natural fibers for material recycling. J. Mater. Cycles Waste Manag..

[33] Li R., Chen S., Yang W., Lin H., Wu Y. (2025). Dual Interfacial Engineering of Glass Fiber-Reinforced Composites: Synergistic Enhancement via Hyperbranched Polyester-Modified Resin and Rigid/Flexible Fiber Coatings. Polym. Compos..

[34] Senthil Kumar J., Thamizhvalavan P., Balasubramanian M., Rajkumar S. (2025). Enhanced mechanical performance and failure mechanisms of woven glass fiber-reinforced polymer composites with optimized multi-walled carbon nanotube reinforcement. Polym. Compos..

[35] Zhou Z., Zhang Z., Huang J., Wang Y. (2024). Water-based intumescent fire resistance coating containing organic-modified glass fiber for steel structure. J. Clean. Prod..

[36] Zhang T., Wang K., Lin B., Yao Y. (2024). The enhancement mechanism of modified basalt fiber on the performance of geopolymer concrete. Constr. Build. Mater..

[37] Zhang X., Xue W., Yang X., Shaikh F.U.A. (2025). Effect of surface treatments on the physical mechanical properties and interfacial microstructure of wood fiber-reinforced geopolymer composites. Ind. Crop. Prod..

[38] Li F., Chen D., Yang Z., Lu Y., Zhang H., Li S. (2022). Effect of mixed fibers on fly ash-based geopolymer resistance against carbonation. Constr. Build. Mater..

[39] Ni S., Luo W., Wang Z. (2024). Investigating the influence of acid-base/KH550 composite surface modified BF on the properties of fiber-reinforced SBS-modified asphalt mastic. Constr. Build. Mater..

[40] Gunwant D. (2024). Moisture resistance treatments of natural fiber-reinforced composites: a review. Compos. Interfaces.

[41] Liu J., Yue W.V., Ma S., Ding W., Yue Z.Q. (2025). Accurate determination of clay contents in Shanghai soils. J. Rock Mech. Geotech. Eng..

[42] Liu C.Y., Ku C.Y., Wu T.Y., Ku Y.C. (2024). An advanced soil classification method employing the random forest technique in machine learning. Appl. Sci..

[43] Shen L., Zhu T., Shi L., Zhao G., Zhang W., Jian X., Xu J. (2025). Dynamic damage evolution and thermal-strain rate coupled modeling of CF/PPESK composites: synergistic coupling of thermal activation and hysteresis mechanisms. Compos. B Eng..

[44] Ma P., Hu D., Liu X., Wang Y., Pan J., Wang G., Deng J., Wang R. (2025). Temperature dependent fatigue damage evolution of SiCf/SiC composites captured using in-situ X-ray imaging and strain analysis. Compos. Appl. Sci. Manuf..

[45] Albayati A.H., Al-ani A.F., Mohammed A.M., Al-Kheetan M.J., Moudhafar M.M., Jweihan Y.S. (2025). Understanding the effectiveness of elastomeric and plastomeric polymers on the high-temperature performance of asphalt binders. Innov. Infrastruct. Solut..

[46] Weise K., Ukrainczyk N., Koenders E. (2023). Pozzolanic reactions of metakaolin with calcium hydroxide: review on hydrate phase formations and effect of alkali hydroxides, carbonates and sulfates. Mater. Des..

[47] Weise K., Ukrainczyk N., Koenders E. (2024). Pozzolanic metakaolin reactions: stoichiometric and kinetic modeling. Mater. Des..

[48] Wang S., Lang L., Wei M., He X., Wang R., Yu C., Feng S., Niu Z., Ma H. (2022). Strength and microstructural characteristics of cement-solidified salt-rich dredged silt modified by nanoparticles. Mar. Georesour. Geotechnol..

[49] Zhao Y., Guo Y., Sun Y., Zhou X., Min Z., Lin Q., Chen S., Li Y., Jiang M., Feng A., Kang S. (2025). Mechanical and microstructural properties of glass powder-modified recycled brick-concrete aggregate concrete. Case Stud. Constr. Mater..

[50] Chen S., Ou X., Jiang J., Tan Z. (2023). Experimental Study on the Curing Mechanism of Red Mud-Based Stabilized Soil Co-Modified by Nano-SiO2 and Gypsum. Materials.

[51] Atiq Orakzai M. (2021). Hybrid effect of nano-alumina and nano-titanium dioxide on mechanical properties of concrete. Case Stud. Constr. Mater..

[52] Madhkhan M., Katirai R. (2019). Effect of pozzolanic materials on mechanical properties and aging of glass fiber reinforced concrete. Constr. Build. Mater..

[53] Madhkhan M., Rafiei H.R., Safarkhani M. (2026). Effect of pozzolanic materials on glass fiber reinforced concrete durability in freeze-thaw cycles. Results Eng..

[54] Xu Y., Ma Y., Xiao C., Zhang Y., Gan L., Lin D., Gao H. (2026). Optimization of mechanical properties of modified rammed earth materials using cement-aggregate-fiber composites: an experimental study. Discov. Civ. Eng..

[55] Jin L., Li S., Chen X., Hu M., Geng Y., Sui S., Hu Q., Liu Y., Li K. (2024). Effect and mechanisms of surface treatment using nano titanium dioxide on the permeability of cementitious materials. J. Build. Eng..

[56] Bunea G., Alexa-Stratulat S.M., Mihai P., Toma I.O. (2023). Use of clay and titanium dioxide nanoparticles in mortar and concrete—a state-of-the-art analysis. Coatings.

[57] Muhammad M., Ma R., Du A., Fan Y., Zhao X., Cao X. (2023). Preparation and Modification of Polydopamine Boron Nitride—Titanium Dioxide Nanohybrid Particles Incorporated into Zinc Phosphating Bath to Enhance Corrosion Performance of Zinc Phosphate-Silane Coated Q235 Steel. Materials.

[58] Feng S., Guan S. (2025). Influence of nano-SiO2 and nano-TiO2 on early hydration process of cement: Hydration rate, hydration products microstructure, calcium ion solubility, and diffusion ability. Constr. Build. Mater..

[59] Zhang T., Wang Z., Wang W., Li G., Guo H., Zhang H. (2025). Property enhancement and mechanism of cement-based composites from the perspective of nano-silica dispersion improvement. Case Stud. Constr. Mater..

[60] Yu X., Wu X., Zhu P., Liu C., Qiu C., Cai Z. (2025). Mechanism of Strength Degradation of Fiber-Reinforced Soil Under Freeze–Thaw Conditions. Buildings.

[61] Feng P., Ma L., Zhang M., Quan Y., Li M., Zhou X., Liu X., Jian X., Xu J. (2024). Constructing a novel moderately modulus “rigid-flexible” structure with synergistic reinforcement on the carbon fiber surface to enhance the mechanical properties of carbon fiber/epoxy composites at elevated temperatures. ACS Appl. Mater. Interfaces.

[62] Liu L., Xiang D., Harkin-Jones E., Liu Z., Lin L., Xie G., Gong Y., Zhao C., Li H., Liu H. (2025). Hierarchically structured basalt fiber reinforced poly (arylene ether nitrile) composites for enhanced mechanical, electrical, and damage self-sensing performance. Compos. Sci. Technol..

[63] Chen H., Zhao C., Zhang R., Xing J., Huang L., Qian Y. (2023). Macro-micro damage model of the effect of freeze–thaw on jointed rocks considering compaction deformation. Eng. Fail. Anal..

[64] Sun W., Ma J., Jin J., Li S., Liu Q., Wang H. (2024). Quantitative study of the failure characteristics of sandstone with freeze–thaw damage: Insight into the cracking behavior. Rock Mech. Rock Eng..

[65] Sun S., Liu X., Liu H., Shi C., Xu L., Huang Z., Sui Y. (2024). Mechanical properties and acoustic emission characteristics of basalt fiber reinforced cemented silty sand subjected to freeze–thaw cycles. Sci. Rep..

[66] Wang Y., Huang X., Guo S., Zhang X., Nie Y. (2024). An experimental investigation on freeze–thaw resistance of fiber-reinforced red mud-slag-based geopolymer. Case Stud. Constr. Mater..

[67] Kang D., Jia S., Zhao C., Ni Y., Qi J., Kang Z., Sui Y., Wei F., Xiao B., Meng Q. (2024). High-temperature resistance performance of silica aerogel composites through fiber reinforcement. Ceram. Int..

[68] Zhen R., Jiang Y.S., Li F.F., Xue B. (2017). A study on the intercalation and exfoliation of illite. Res. Chem. Intermed..

[69] Kotler J.M., Quinn R.C., Foing B.H., Martins Z., Ehrenfreund P. (2011). Analysis of mineral matrices of planetary soil analogues from the Utah Desert. Int. J. Astrobiol..

[70] Deon F., van Ruitenbeek F., van der Werff H., van der Meijde M., Marcatelli C. (2022). Detection of interlayered illite/smectite clay minerals with XRD, SEM analyses and reflectance spectroscopy. Sensors.

[71] Li K., Kong S., Liang Y., Ali M., Zhang Y., Zhao Y. (2023). Geochemical and Microstructural Characteristics of Clay Minerals and Their Effects on the Pore Structure of Coal-Measure Shale: A Case Study in Qinshui Basin, China. Energies.

[72] Hunger A., Carl G., Rüssel C. (2010). Formation of nano-crystalline quartz crystals from ZnO/MgO/Al2O3/TiO2/ZrO2/SiO2 glasses. Solid State Sci..

[73] Wei T., Chunran W., Yuanyuan M., Shicong K., Baojian Z., Feng X., Baojun Z. (2025). A comparative study of dry and wet carbonation treatment on the recycled mortar powder: Material characteristics, hydration kinetics and sustainability. Constr. Build. Mater..

[74] Yang J., Li Z., Yang C., Gu X., Zhao J. (2024). Study on the activation and pozzolanic reaction mechanism of lithium slag under the effect of composite activation. Constr. Build. Mater..

[75] Wang W., Kang H., Li N., Guo J., Girma D.Y., Liu Y. (2022). Experimental investigations on the mechanical and microscopic behavior of cement-treated clay modified by nano-MgO and fibers. Int. J. GeoMech..

[76] Bhullar S., Goyal N., Gupta S. (2022). Synthesizing and optimizing Rutile TiO2 nanoparticles for magnetically guided drug delivery. Int. J. Nanomed..

[77] Du Y., Niu X., Li W., Liu J., Li J. (2022). Synthesis of high-energy faceted TiO2 nanocrystals with enhanced photocatalytic performance for the removal of methyl orange. Catalysts.

[78] Lokesh K.S., Ramachandra C.G., Kumar T.P., Kanti P.K., Paramasivam P., Ayanie A.G. (2025). Effect of bonding characteristics of major constituents of mineral filler-based glass fiber reinforced with epoxy composites. Sci. Rep..

[79] Sherif G., Chukov D.I., Tcherdyntsev V.V., Stepashkin A.A., Zadorozhnyy M.Y., Shulga Y.M., Kabachkov E.N. (2024). Surface treatment effect on the mechanical and thermal behavior of the glass fabric reinforced polysulfone. Polymers.

[80] Indumathy B., Sathiyanathan P., Prasad G., Reza M.S., Prabu A.A., Kim H. (2023). A comprehensive review on processing, development and applications of organofunctional silanes and silane-based hyperbranched polymers. Polymers.

[81] Aziz T., Ullah A., Fan H., Jamil M.I., Khan F.U., Ullah R., Iqbal M., Ali A., Ullah B. (2021). Recent progress in silane coupling agent with its emerging applications. J. Polym. Environ..

[82] Zhu D., Kurahashi E., You H., Wada T., Chammingkwan P., Taniike T. (2022). Enhancing mechanical properties of graft-type nanocomposites using organically modified SiO2 and polypropylene containing reactive methoxy groups. Polymers.

[83] Soltani A., O’Kelly B.C., Horpibulsuk S., Taheri A. (2024). Unique relationship between optimum compaction properties of fine-grained soils across rational compactive efforts: A validation study. Transp. Infrastruct. Geotechnol..

[84] Zhang Q., Zhang X., Wang L., Zhang S. (2025). Synthesis, Characterization, and Cementitious Activity of the Magnesium Silicate Hydrate and Calcium Silicate Hydrate from Coal Gangue. Molecules.

[85] Gao X., Hou L., Yang W., Dong L., Ge X. (2025). Lignin-based nanoparticles stabilized Pickering emulsion for enhanced catalytic hydrogenation. Langmuir.

[86] Henderson G.S., Bancroft G.M., Nesbitt H.W., Dean P.A.W., Cicconi M.R. (2025). The high frequency Raman bands of vitreous SiO2. Phys. Chem. Glasses: Eur. J. Glass Sci. Technol. B.

[87] Kshirsagar D.V., Joshi G.M. (2025). Epoxy–melamine–cenosphere hybrid composites: complete analysis for engineering applications. Polym. Int..

[88] Qian C., Li M.J., Wang R. (2025). Self-sensitized brown-TiO2 photocatalyst for highly efficient CO2 reduction under ambient conditions. Fuel.

[89] Yang M., Chen S., Zhang Z., Cheng L., Zhao J., Bai R., Wang W., Gao W., Yu W., Jiang X., Yan X. (2024). Stimuli-responsive mechanically interlocked polymer wrinkles. Nat. Commun..

[90] Wang G., Zou Z., Liang G., Ye T., Ma S., Chen Y., Lv G. (2025). Interplay of clay mineral structures and PDDA functionalization in enhancing perchlorate adsorption for water treatment. J. Alloys Compd..

[91] Li Q., Shi W., Yang Q. (2021). Method to determine van der Waals potential energy of particle interactions for soil clay by dynamic light scattering. Eur. J. Soil Sci..

[92] Lei B., Zhang X., Fan H., Wu S., Zhao C., Ni W., Liu C. (2025). A Strategy to Optimize the Mechanical Properties and Microstructure of Loess by Nano-Modified Soil Stabilizer. Materials.

[93] Lu J., Deng F., Pei W., Wan X., You Z., Zhang Z. (2024). Mitigating frost heave and enhancing mechanical performance of silty clay with sisal fibre and geopolymer. Constr. Build. Mater..

